# Mural Cell SRF Controls Pericyte Migration, Vessel Patterning and Blood Flow

**DOI:** 10.1161/CIRCRESAHA.122.321109

**Published:** 2022-07-14

**Authors:** Michael M. Orlich, Rodrigo Diéguez-Hurtado, Regine Muehlfriedel, Vithiyanjali Sothilingam, Hartwig Wolburg, Cansu Ebru Oender, Pascal Woelffing, Christer Betsholtz, Konstantin Gaengel, Mathias Seeliger, Ralf H. Adams, Alfred Nordheim

**Affiliations:** 1 Department of Molecular Biology, Interfaculty Institute for Cell Biology, University of Tuebingen, Germany (M.M.O., C.E.O., P.W., A.N.).; 2 International Max Planck Research School (IMPRS) “From Molecules to Organisms,” Tuebingen, Germany (M.M.O., A.N.).; 3 Institute for Ophthalmic Research, Centre for Ophthalmology (R.M., V.S., M.S.), University Clinic Tuebingen (UKT), Germany.; 4 Department of General Pathology and Pathological Anatomy, Institute of Pathology and Neuropathology (H.W.), University Clinic Tuebingen (UKT), Germany.; 5 Department of Immunology, Genetics and Pathology, Rudbeck Laboratory, Uppsala University, Sweden (M.M.O., C.B., K.G.).; 6 Department of Tissue Morphogenesis, Max Planck Institute for Molecular Biomedicine, Muenster, Germany (R.D.-H., R.H.A.).; 7 Faculty of Medicine, University of Muenster, Muenster, Germany (R.D.-H., R.H.A.).; 8 Now with Rudbeck Laboratory C11, Dag Hammarskjölds Väg 20, 751 85 Uppsala, Sweden (M.M.O.)

**Keywords:** endothelial cells, mice, muscle, smooth, vascular, pericytes, serum response factor

## Abstract

Pericytes and vascular smooth muscle cells, collectively known as mural cells, are recruited through PDGFB (platelet-derived growth factor B)-PDGFRB (platelet-derived growth factor receptor beta) signaling. MCs are essential for vascular integrity, and their loss has been associated with numerous diseases. Most of this knowledge is based on studies in which MCs are insufficiently recruited or fully absent upon inducible ablation. In contrast, little is known about the physiological consequences that result from impairment of specific MC functions. Here, we characterize the role of the transcription factor SRF (serum response factor) in MCs and study its function in developmental and pathological contexts.

**Methods:**

We generated a mouse model of MC-specific inducible *Srf* gene deletion and studied its consequences during retinal angiogenesis using RNA-sequencing, immunohistology, in vivo live imaging, and in vitro techniques.

**Results:**

By postnatal day 6, pericytes lacking SRF were morphologically abnormal and failed to properly comigrate with angiogenic sprouts. As a consequence, pericyte-deficient vessels at the retinal sprouting front became dilated and leaky. By postnatal day 12, also the vascular smooth muscle cells had lost SRF, which coincided with the formation of pathological arteriovenous shunts. Mechanistically, we show that PDGFB-dependent SRF activation is mediated via MRTF (myocardin-related transcription factor) cofactors. We further show that MRTF-SRF signaling promotes pathological pericyte activation during ischemic retinopathy. RNA-sequencing, immunohistology, in vivo live imaging, and in vitro experiments demonstrated that SRF regulates expression of contractile SMC proteins essential to maintain the vascular tone.

**Conclusions:**

SRF is crucial for distinct functions in pericytes and vascular smooth muscle cells. SRF directs pericyte migration downstream of PDGFRB signaling and mediates pathological pericyte activation during ischemic retinopathy. In vascular smooth muscle cells, SRF is essential for expression of the contractile machinery, and its deletion triggers formation of arteriovenous shunts. These essential roles in physiological and pathological contexts provide a rationale for novel therapeutic approaches through targeting SRF activity in MCs.

**Novelty and Significance**

**What Is Known?**

- Pericytes and vascular smooth muscle cells (vSMCs) collectively known as mural cells cover endothelial cells which form the inner lining of blood vessels.
- Pericytes are essential to maintain endothelial barrier function and their loss is associated with numerous diseases.
- vSMCs regulate blood flow, but it is not known to what extent changes in blood flow influence blood vessel patterning.

**What New Information Does This Article Contribute?**

- PDGFB (platelet-derived growth factor B)-PDGFRB (platelet-derived growth factor receptor beta) signaling activates the SRF (serum response factor) transcription factor via its cofactor MRTF (myocardin-related transcription factor) to promote pericyte migration.
- *Srf* deletion in mural cells results in altered pericyte and vSMC morphology, defects in the actin cytoskeleton, and reduced pericyte migration.
- Blockade of SRF signaling in pericytes under ischemic conditions mitigates pathological angiogenesis, making SRF a potential drug target in ischemic retinopathies.
- In vSMCs, SRF controls the expression of contractile genes, and its deletion leads to severe blood vessel patterning defects and the formation of arteriovenous shunts, which in turn, cause a redirection of retinal blood flow that leaves part of the capillary network poorly perfused.

Pericytes and vSMCs, collectively called mural cells are found in close contact with endothelial cells in all blood vessels. Pericytes line capillaries and venules, whereas vSMCs encircle arteries, arterioles, and veins. During angiogenesis, endothelial cells actively recruit pericytes by secretion of the growth factor PDGFB. Recruited pericytes provide stabilization to the immature vasculature. vSMCs express a battery of contractile proteins that maintain and dynamically regulate vascular tone and blood flow. By knocking out the transcription factor SRF specifically in mural cells, we found that pericytes develop a compromised capacity to migrate during angiogenesis. Under ischemic conditions, mural SRF is overactive, causing pathological behavior of pericytes. Inhibition of SRF signaling might therefore be a potential treatment for ischemic retinopathies. vSMCs lacking SRF show a different phenotype: they lose expression of contractile proteins, causing failure of vascular tone regulation. Lack of vascular tone leads to severe patterning defects in the developing retinal vasculature, ultimately forcing the formation of arteriovenous shunts. Those shunts show a redirection of blood flow leaving large parts of the capillaries undersupplied. Our work unravels distinct functions for SRF in pericytes and VSMCs and illustrate the critical importance of both pericytes and VSMCs for vascular patterning.

**In This Issue, see p 287**

**Meet the First Author, see p 288**

Blood vessels are composed of endothelial cells (ECs) lining the vascular lumen and surrounding mural cells (MCs).^1^ MCs include pericytes, which cover capillaries, the smallest diameter blood vessels, and vascular smooth muscle cells (vSMCs),^2^ which cover arteries, arterioles, venules, and veins, the larger caliber vessels.^3^

During angiogenesis, new blood vessels develop from preexisting ones by vascular sprouting. This process involves endothelial tip cells, specialized ECs that temporarily adopt a motile phenotype and extend numerous filopodia. Tip cells spearhead the sprouting vessels and secrete PDGFB (platelet-derived growth factor B), thereby attracting pericytes that express the corresponding PDGFRB (PDGF receptor beta), to comigrate along the nascent vessel sprout. Pericyte recruitment is generally considered to aid vessel stabilization, and numerous studies have demonstrated the importance of pericytes for vascular function.^4^ In the brain, pericytes have been shown to express high levels of transporters and are considered to play a crucial role in maintaining homeostasis at the neurovascular unit.^3,5^ In accordance, failure to recruit pericytes results in impaired formation of the blood-brain barrier and the blood-retina barrier.^3^ Loss of pericyte coverage has been identified as an early event in diabetic retinopathy, which is associated with breakdown of the blood-retina barrier.^6^ Most of our knowledge about pericyte function comes from studies in which pericyte recruitment has been compromised, or in which pericytes have been ablated. Only few studies have investigated the physiological consequences that arise if specific aspects of pericyte biology or functions are inactivated.^7–10^ Pericyte research is further complicated by the fact that identification and characterization of pericytes is challenging due to the lack of unambiguous markers. Commonly used markers to identify pericytes, such as PDGFRB, NG2 (neural/glial antigen 2), DES (desmin), or CD13, are also expressed by other cell types and therefore accurate identification of pericytes requires, in addition, attention to their localization and morphology.^11^

In contrast to pericytes, vSMCs are highly contractile and express a specific set of smooth muscle genes (SMG). Through a basal level of constriction, vSMCs create the vascular tone, which is essential to direct blood flow into capillaries.^5^ Vascular tone differs between organs and is dependent on the balance of competing vasoconstrictors and vasodilators.^12^ Dysregulation of vSMC contractility is associated with hypertension, aortic stiffness, and chronic venous disease.^13–15^

SRF (serum response factor) is a conserved, ubiquitously expressed transcription factor that belongs to the MADS-box (Minichromosome Maintenance Factor 1‚ Agamous‚ Deficiens‚ Serum Response Factor) protein family and is known to regulate motile functions in a variety of cell types.^16–18^ SRF is activated either by Rho/actin or Ras/MAPK (rat sarcoma/mitogen-activated protein kinase) signaling.^17,18^ Those pathways involve different cofactor proteins, either MRTF (myocardin-related transcription factor) or TCFs (ternary complex factor) and result in the expression of distinct sets of target genes. SRF has been reported to drive, among others, the expression of SMGs alpha-smooth muscle actin (*Acta2*, αSMA) and transgelin (*Tagln*, SM22α [smooth muscle 22 alpha]) in visceral SMCs,^16,19^ but its role in pericytes and SMCs of the vasculature has not been thoroughly investigated. To address this question, we used *Pdgfrb-CreER*^T2^ mice, which allow to delete SRF in pericytes and SMCs of the vasculature^7,20,21^ and, importantly, prevent the lethality associated with SRF deletion in visceral SMCs.^19,22^

We demonstrate that SRF is crucial for distinct functional roles in pericytes and vSMCs, respectively. In pericytes, SRF is essential for cell migration downstream of PDGFRB signaling and mediates the pathological activation of pericytes during ischemic retinopathy. In vSMCs, SRF is essential for the expression of SMC genes, and its deletion triggers the formation of arteriovenous malformations (AVMs).

## Methods

### Data Availability

A The data that support the findings of this study are available from the corresponding author upon reasonable request. The bulk raw sequencing data and the processed counts table of our study are available at the NCBI Gene Expression Omnibus (GSE205491). A detailed description of the methods is provided as Supplemental Material.

## Results

### Conditional MC-Specific Deletion of *Srf*

**Figure 1.**
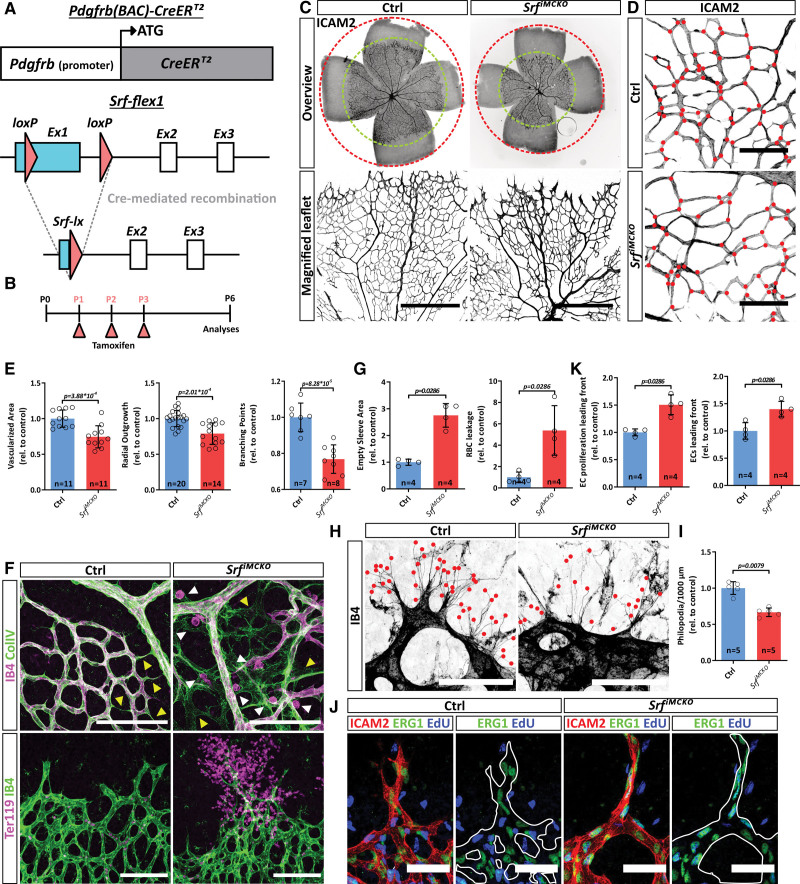
**SRF (serum response factor)-deficient pericytes (PCs) impair early vascular morphogenesis of the retina. A**, Schematic representation of the Pdgfrb(BAC)-CreER^T2^ transgene and Cre-mediated recombination of the Srf-flex1 (Floxed exon 1) allele. **B**, Tamoxifen administration regime and time point of analyses. **C**, Epifluorescence overview images (**upper**) and confocal images (**lower**) of whole-mount retinas stained for ICAM2 (intercellular adhesion molecule 2) to visualize vascularization and vascular outgrowth of the primary vascular plexus. Scale bar, 250 µm. **D**, Comparison of vascular branch points in control (Ctrl) and *Srf^iMCKO^* retinas. Red dots indicate branch points. Scale bar, 100 µm. **E**, **G**, **and K**, Quantifications of morphometric parameters, pruning, leakage, and endothelial cell (EC) proliferation. **F**, Confocal images of vascular pruning events visualized by empty COLIV (Collagen IV) sleeves (**upper**; yellow arrowheads) and vascular leakage, visualized as isolectin B4 (IB4)–positive immune-cells extravasation (**upper**; white arrowheads) and Ter119 positive red blood cell extravasation (**lower**). Scale bar, 100 µm. **H** and **I**, High-resolution confocal images of tip cell filopodia at the sprouting front (stained by IB4, marked by red dots) and respective quantification. Scale bar, 50 µm. **J**, Confocal images of EC proliferation (5-ethynyl-2′-deoxyuridine [EdU^+^]/ERG1^+^ [ETS-related gene 1]) at the angiogenic front. Scale bar, 50 µm. Error bars show s.d. of the mean. Statistical significance in **E** was determined using the Shapiro-Wilk test for normality followed by an unpaired *t* test with Welch correction (2-tailed). For the data shown in **G**, **K**, and **I** the unpaired Mann-Whitney test (2-tailed) was used. P indicates postnatal day. BAC indicates bacterial artificial chromosome; and rel., values relative to control.

To address the role of SRF in MCs in vivo, we crossed floxed *Srf*^flex1/flex1^ (meaning floxed exon 1, also referred to as *Srf^flox/flox^* mice) mice^20^ with *Pdgfrb-CreER*^T2^ mice, which have been shown to efficiently target MCs.^7,21^ We induced MC-specific deletion of *Srf* (hereafter referred to as *Srf^iMCKO^*) in newborn mice via tamoxifen administration at postnatal days (P) 1 to 3 and focused our analysis on the retinal vasculature. We choose 3 distinct time points (P6, P12, and after 4–8 weeks) for analysis to investigate the role of SRF during vascular sprouting, remodeling, and maturity of the retinal vasculature (Figure 1A and 1B). To monitor recombination specificity and efficiency, we further introduced the *Rosa26^mTmG^* reporter^23^ to the *Pdgfrb-CreER*^T2^::*Srf^flox/flox^* mouse line. The *Rosa26^mTmG^* reporter expresses membrane-targeted GFP in cells, in which Cre-mediated recombination has occurred and revealed that, at P6, around 80% of MCs had recombined the *Rosa26^mTmG^* reporter (Figure S1A through S1C).

Polymerase chain reaction (PCR) analysis of whole retinal lysates further confirmed the presence of a 380 bp long *Srf*-exon 1 PCR product (S*rf-lx*) in *Srf^iMCKO^* mice, indicating successful recombination of the *Srf-flex1* allele. In contrast, in control mice, only a 1.34 kbp long PCR product, corresponding to the nonrecombined LoxP (Locus of Crossover in P1) flanked gene sequence of the *Srf-flex1* allele was amplified (Figure S1D).

Additional gene expression analysis of retinal MC populations sorted via fluorescence-activated cell sorting (FACS), showed efficient downregulation (over 99%) of *Srf* expression (Figure S1E) in *Srf^iMCKO^* mice. Taken together, our conditional gene deletion approach allowed for successful and reliable deletion of *Srf* in MCs.

### SRF Function in Pericytes Is Crucial for Normal Development of the Retinal Vasculature

To investigate the effects of mural *Srf* deletion on retinal angiogenesis, we analyzed *Srf^iMCKO^* and control retinas at P6. At this stage, the retinal vasculature is still actively sprouting and capillaries at the sprouting front become invested by pericytes. At the same time, the more proximal vascular plexus is remodeling into a hierarchical network with arteries, arterioles, venules, and veins. In *Srf^iMCKO^* retinas, radial vessel outgrowth was delayed and the vascularized area and number of capillary branches were decreased (Figure 1C through 1E). Increased levels of extravasated erythrocytes also suggested reduced barrier properties of the vasculature in *Srf^iMCKO^* retinas (Figure 1F and 1G). We further observed a decrease in the number of tip cell filopodia and a severe dilation of blood vessels at the sprouting front (Figure 1H and 1I). In line with this, 5-ethynyl-2′-deoxyuridine labeling (EdU) of proliferating cells in combination with the EC-specific nuclear marker ERG1 (ETS-related gene 1) revealed an increase in EC proliferation in *Srf^iMCKO^* retinas (Figure 1J and 1K; Figure S1H). Besides the abovementioned effects on the angiogenic sprouting front, the loss of *Srf* in MCs also affected remodeling of the proximal vascular plexus. We observed crossing of arteries and veins, which represents a microvascular abnormality termed nicking and is also observed in the human retina^24^ (Figure S1F and S1G). We also noted abundant deposits of the basement membrane protein collagen IV without attached ECs, so-called empty matrix sleeves (Figure 1F), which indicate excessive vascular pruning.^25^ These observations argue for defective remodeling and instability of nascent vessels in the proximal vascular plexus (Figure 1F and 1G).

### SRF Mediates Pericyte Migration Downstream of PDGFB via Cytoskeletal Rearrangements

**Figure 2.**
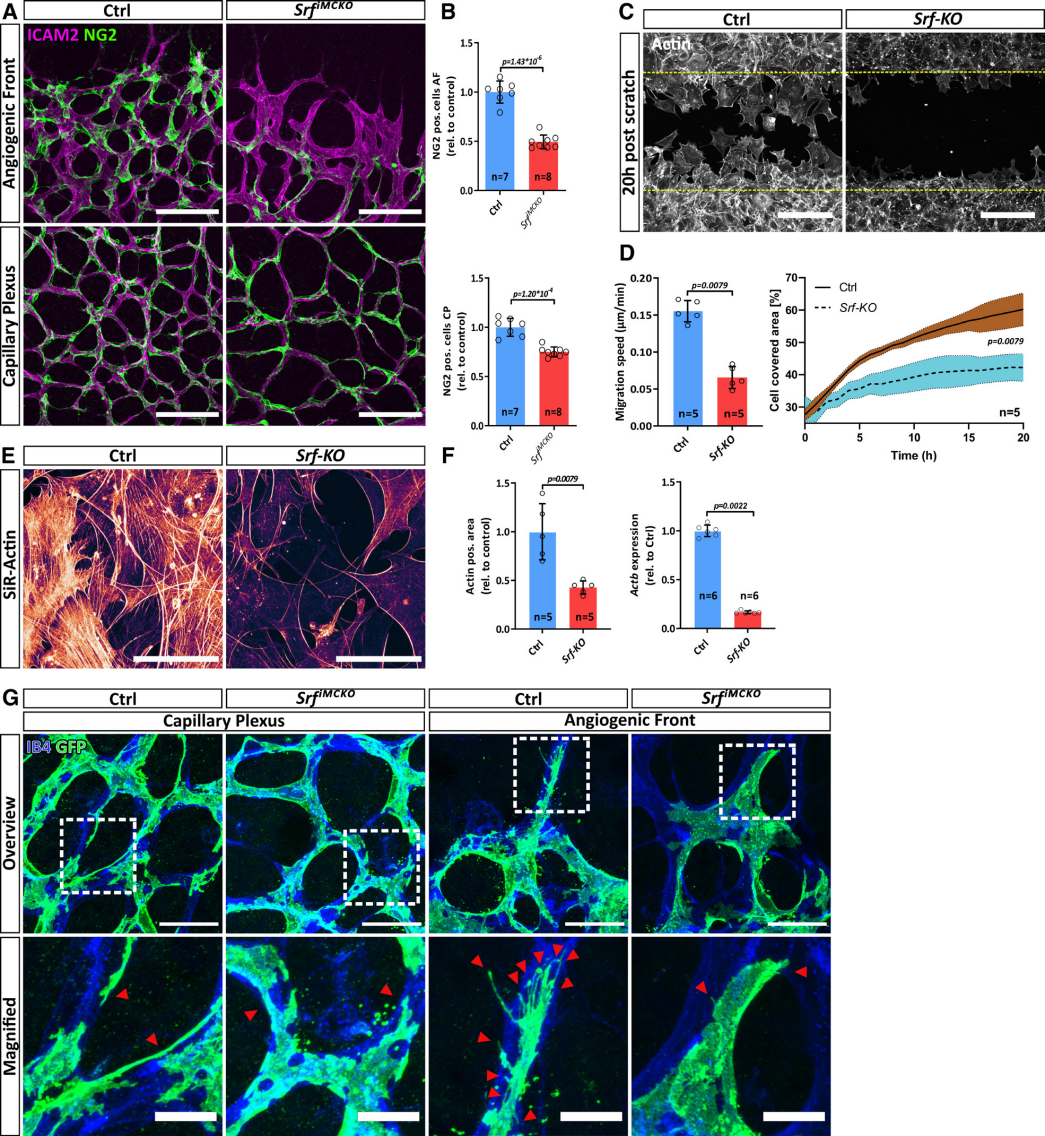
**SRF (serum response factor) controls cytoskeletal homeostasis and migration of pericytes (PCs). A**, Confocal image showing PC coverage (NG2 [neural/glial antigen 2], green) on vessels (ICAM2 [intercellular adhesion molecule 2], magenta) at the angiogenic front (AF; **upper**) and the capillary plexus (CP; **lower**). Scale bar, 100 µm. **B**, Respective quantification of PC coverage. **C and D**, Confocal images of a 20h post scratch wound assay and respective quantifications using control (Ctrl) and *Srf-KO* pericytes from the brain (pBPCs), stained with the F-Actin staining dye silicon-rhodamine (SiR)-Actin. Scale bar, 250 µm. **E**, *Srf-KO* and control pBPC cultures stained with SiR-Actin. Note the reduction of F-actin in *Srf-KO* cells. Scale 100 µm. **F.** Respective quantification of actin positive area and relative transcript levels of *Actb* determined by quantitative polymerase chain reaction. **G**, High-resolution confocal images of PCs labeled by mTmG reporter expression (green) at the CP and the AF. Endothelial cells are stained for isolectin B4 (IB4). Red arrowheads point to tube-like protrusions (in the capillary plexus) or filopodia (in the angiogenic front). Note the absence of filopodia as well as the shortened and flattened protrusions at *Srf^iMCKO^* PCs. Scale bars, 25 µm, 10 µm (magnified). Error bars show SEM. Statistical significance in **B** was determined using the Shapiro-Wilk test for normality followed by an unpaired *t* test with Welch correction (2-tailed). For the data shown in **D** and **F** the unpaired Mann-Whitney (2-tailed) was used. Number of analyzed animals/repetitions (n) is indicated. GFP indicates green fluorescent protein; pos., positive; and rel., values relative to control

Since the vascular defects that we observed shared similarities with mouse models of postnatal pericyte depletion,^7,8,26^ we addressed pericyte recruitment and coverage in *Srf^iMCKO^* retinas. Immunostainings using antibodies against the pericyte markers NG2, DES, and PDGFRB^2^ revealed a 50.6% reduction of pericyte coverage at the angiogenic sprouting front (Figure 2A and 2B; Figure S2A and S2B). Interestingly, vessels at the central plexus only showed a 25.5% reduction of pericyte coverage, suggesting that pericyte migration along newly formed blood vessels was especially affected. In addition, we observed a 25% reduction in pericyte proliferation at the angiogenic front but not at the capillary plexus in *Srf^iMCKO^* retinas (Figure S2C and S2D). Immunostainings for cleaved-caspase3 did not reveal any differences in cell survival (Figure S2E and S2F).

To specifically address, if pericyte migration is compromised upon *Srf* deletion, we isolated primary pericytes from the brain (pBPCs) of *Srf-flex1* and control mice and performed migration assays. To delete *Srf* in pBPC cultures, we exposed these cells to Tat-Cre, a membrane-permeable version of the Cre recombinase. Subsequent quantitative PCR analysis showed that this approach led to an over 99% reduction of *Srf* mRNA and Western blot analysis confirmed the loss of the full-length SRF protein (Figure S2G and S2H). In *Srf-KO* pBPCs only a truncated and nonfunctional version of the SRF protein is detected at ≈36 kDa.^19^ In scratch wound assays, *Srf* deleted pBPC cultures (hereafter referred to as *Srf-KO*) showed a significant reduction in collective cell migration, as well as in the speed of individual migrating cells (Figure 2C and 2D, Video S1). Likewise, we also observed reduced migration of *Srf-KO* cells in a transwell assay (Figure S2I and S2J).

Since SRF is known to regulate cellular motility through transcriptional control of genes encoding regulators of actin dynamics in other cell types (reviewed in Olson & Nordheim, 2010),^18^ we used the membrane-permeable F-actin staining dye silicon-rhodamine (SiR)-Actin,^27^ to visualize actin dynamics in living cells, and performed a series of time-lapse experiments. These experiments revealed a substantial reduction of F-actin in *Srf-KO* cells (Figure 2E and 2F). In accordance, we also observed a significant reduction of beta-actin gene (*Actb*) expression in *Srf-KO* cells (Figure 2F). We further tested to which degree SRF-mediated cytoskeletal rearrangements are required downstream of PDGFB for pericyte migration. We, therefore, treated starved pBPCs with PDGFB and live imaged the changes in actin dynamics using SiR-Actin. Upon PDGFB stimulation, control pBPCs showed intensified actin dynamics and increased cell motility. In contrast, *Srf-KO* cells displayed almost no reaction to PDGFB stimulation (Figure S2K; Video S2). Taken together, these results indicate that the observed migration defects of *Srf^iMCKO^* pericytes are caused by the inability of the actin cytoskeleton to respond to the natural PDGFB gradient originating from endothelial tip cells.

To investigate pericyte morphology in vivo, we crossed the *Rosa26^mTmG^* reporter into the *Srf^iMCKO^* background and subsequently analyzed labeled pericytes in *Pdgfrb-CreER*^T2^::*Rosa26^mTmG^*::*Srf-flex1/Srf-flex1* (*Srf^iMCKO^*) and *Pdgfrb-CreER*^T2^::*Rosa26^mTmG^*::*Srf-flex1/wt* littermate control retinas. In the absence of tamoxifen, the *Rosa26^mTmG^* reporter ubiquitously expresses tdTomato. However, upon tamoxifen induction, Cre-mediated recombination leads to expression of membrane tagged eGFP (enhanced green fluorescent protein), which reliably outlines cell morphology (Figure 2G). We found that control pericytes at the capillary plexus attached tightly to the endothelium and extended numerous thin protrusions that connected pericytes with each other. In contrast, *Srf^iMCKO^* pericytes displayed an overall less ramified morphology and only formed short and stubby protrusions. At the angiogenic front, morphological changes were even more pronounced. We noticed that control pericytes often extended filopodia, which were oriented towards the angiogenic sprouting front, suggesting that pericytes might use filopodia, similarly to ECs, for migration and to sense the PDGFB gradient (Figure 2G). In contrast, SRF-deficient pericytes had entirely lost the ability to form filopodia, appeared partially detached from ECs, and had adopted an abnormal cell morphology (Figure 2G). The inability of *Srf^iMCKO^* pericytes to form filopodia is in line with the actin remodeling defects that we observed in our *in vitro* experiments and consistent with SRF function in ECs where it also regulates filopodia formation.^28,29^ Taken together, our results suggest a crucial role for SRF in pericyte migration via regulation of actin dynamics.

### PDGFB Signaling Activates SRF via MRTF Cofactors

**Figure 3.**
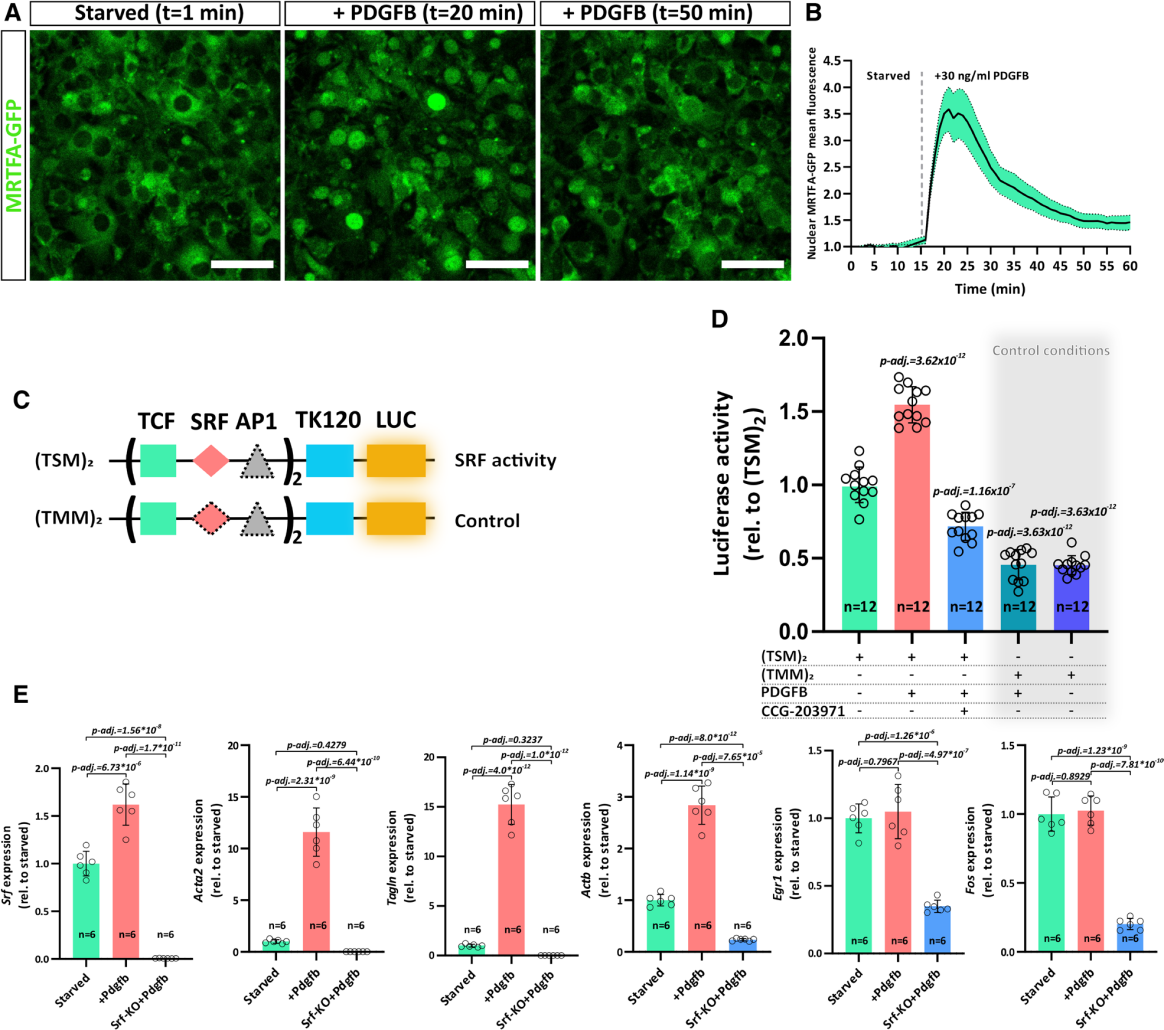
**PDGFB (platelet-derived growth factor B) signals towards SRF (serum response factor) via MRTF (myocardin-related transcription factor) cofactors. A**, Confocal live imaging of 3T3 cells stably transfected with MRTFA-GFP (green fluorescent protein) (green). The cells are shown before and after PDGFB stimulation. Time points are indicated. Scale bar, 50 µm. **B**, Quantification of nuclear mean intensity of MRTFA-GFP signal over the time course of the experiment. In total, 29 cells were quantified. The experiment was carried out three times (n=3). SEM is indicated in green. **C**, Vector constructs used for luciferase assay, containing TCF (ternary complex factor), SRF, and AP1 (activator protein 1)-binding sites. Thymidine kinase (TK) minimal promoter (TK120) drives basal LUC (luciferase) expression. Symbols with dashed borders indicate mutated (unfunctional) binding sites. **D**, Luciferase assay to verify SRF-driven luciferase activity upon PDGFB stimulation. Activity was modulated using vector constructs (TSM)_2_ and (TMM)_2_ as well as by using the MRTF inhibitor CCG-203971. **E**, Gene expression analysis using quantitative polymerase chain reaction (qPCR) of MRTF-SRF target genes (*Acta2*, *Tagln*, and *Actb*) as well as TCF-SRF target genes (*Ehr1* [early growth response 1] and c-*Fos* [Fos proto-oncogene]) in starved and PDGFB stimulated *Srf*-wild type (green and red bars) and *Srf-KO* (blue bars) pericytes from the brain (pBPCs). Error bars indicate SD of the mean. Statistical significance in **D** and **E** was determined using the Shapiro-Wilk test for normality and a one-way ANOVA multiple comparisons test with Tukey-correction (10 comparisons in **D** and 3 in **E**). Number of analyzed repetitions (n) is indicated. KO indicates knockout; and rel.‚ values are relative to control.

The migration defects of SRF-depleted pericytes implied a direct role for SRF downstream of PDGFB signaling in pericyte migration. Most SRF-mediated motility responses have been reported to be regulated via RhoGTPase signaling. RhoGTPase activity stimulates F-actin polymerization, which depletes the cellular G-actin pool. Cytosolic G-actin can bind to MRTFA and MRTFB (myocardin-related transcription factors A and B) and thereby inhibiting nuclear translocation of MRTFs. Increased F-actin polymerization diminishes cytosolic G-actin levels, thereby enabling nuclear translocation of MRTFA and MRTFB and subsequent activation of SRF-directed transcription.^17^ To test if PDGFB also leads to SRF activation via MRTF cofactors, we took advantage of a 3T3 cell line expressing GFP-tagged MRTFA-protein.^30^ To observe potential MRTFA-GFP nuclear translocation upon PDGFB stimulation, we starved these cells overnight to cause MRTFA-GFP to predominantly localize in the cytoplasm (Figure 3A). We subsequently imaged the starved cells using time-lapse-microscopy and, after 15 minutes, stimulated with PDGFB (Video S3). Strikingly, PDGFB stimulation led to a 3.5-fold increase in MRTFA-GFP nuclear translocation already within 5 minutes (T=20 minutes, Figure 3A and 3B) and remained at high levels for another 5 minutes (T=25 minutes), before gradually shifting back to the cytoplasm. Thirty-five minutes after stimulation (T=50 minutes, Figure 3A and 3B), the nuclear MRTFA-GFP signal was reduced to 1.5-fold compared to prestimulation conditions and remained at this level for the rest of the experiment.

To investigate if PDGFB-induced nuclear MRTF translocation results in activation of SRF target genes, we performed luciferase-based reporter assays with promoter sequences containing functional (TSM)_2_ or mutated (TMM)_2_ SRF binding sites (Figure 3C).^28^ PDGFB stimulation of 3T3 cells transiently transfected with the (TSM)_2_ reporter significantly increased basal luciferase activity (Figure 3D). Addition of the MRTF inhibitor CCG-203971 abrogated the PDGFB-induced luciferase activation, demonstrating that PDGFB activates SRF-mediated transcription primarily via MRTF cofactors. The (TMM)_2_ construct served as negative control in those experiments, as SRF cannot bind to the mutated promoter sequence and thus is unable to activate the transcription of target genes. In accordance, PDGFB stimulation does not increase Luciferase activity in 3T3 cells transiently transfected with the (TMM)_2_ construct. To this end, quantitative PCR analysis of PDGFB stimulated pBPCs further indicated a strong induction of the MRTF-SRF target genes *Actb*, *Acta2*, and *Tagln* (Figure 3E). Interestingly, we did not observe the induction of immediate early gene response genes *Egr1* and *c-Fos*, which are dependent on TCF-mediated SRF activation. Taken together, our results strongly suggest that PDGFB-dependent SRF activation and transcription of target genes are mediated via MRTF cofactors.

### SRF Is a Key Determinant of Pathological Pericyte Activation

**Figure 4.**
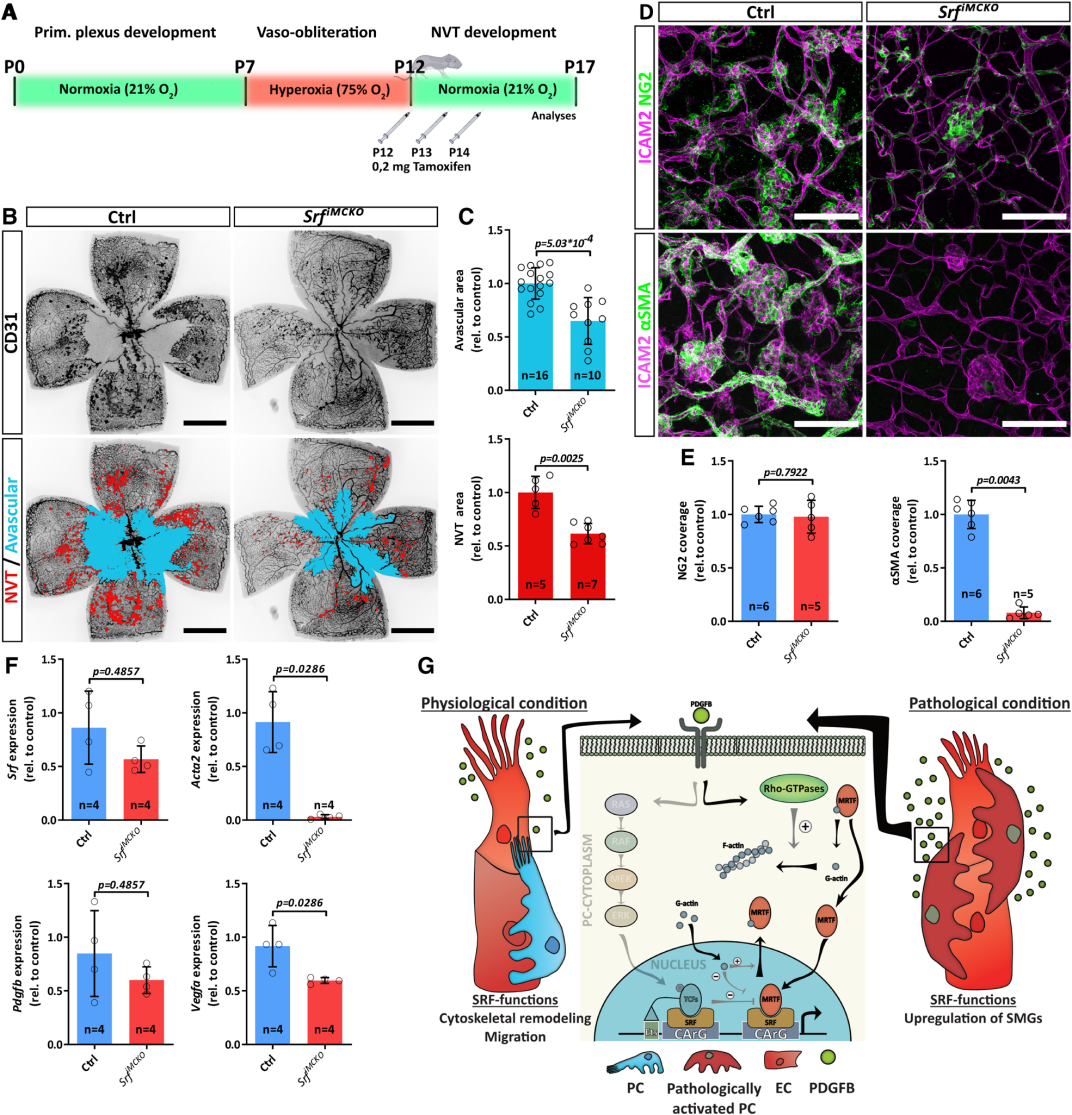
**SRF (serum response factor) is a key determinant of pathological pericyte (PC) activation. A**, Experimental outline of oxygen-induced retinopathy (OIR) experiments. **B**, Epifluorescence overview images of *Srf^iMCKO^* and control (Ctrl) OIR retinas. The avascular (blue) and neovascular tuft (NVT) area (red) are highlighted in the **lower**. Note the reduction of avascular and NVT area in *Srf^iMCKO^* retinas. Scale bars, 1 mm. **C**, Quantification of avascular (**upper** graph) and NVT area (**lower** graph). **D**, High-resolution confocal images of NVTs in control and Srf^iMCKO^ OIR retinas stained for NG2 (neural/glial antigen 2; green, first row), αSMA (alpha-smooth muscle actin; green, second row), and the endothelial cell (EC) marker ICAM2 (intercellular adhesion molecule 2; magenta, first row). Scale bar, 100 µm. **E**, Quantification of PC coverage by NG2 staining as well as pathologically activated PCs by αSMA staining. **F**, Quantification of relative transcript levels of *Srf, Acta2, Pdgfb*, and *Vegfa* (vascular endothelial growth factor A) determined by quantitative polymerase chain reaction from whole OIR retina lysates. **G**, Mechanistic model of SRF guided PC functions under physiological and pathological conditions. Error bars show SD of mean. Statistical significance in **C** (**upper** graph) was determined using the Shapiro-Wilk test for normality followed by an unpaired *t* test with Welch correction (2-tailed). Data shown in **C** (**lower** graph), **E**, and **F** were statistical compared using the unpaired Mann-Whitney test (2-tailed). Number of analyzed animals (n) is indicated. ERK indicates extracellular signal-regulated kinases; MEK‚ MAPK kinase; MRTF‚ myocardin-related transcription factor; P, postnatal days; PDGFB, platelet-derived growth factor B; Prim.‚ primary; RAF‚ rat fibrosarcoma; RAS, rat sacoma; rel.‚ values relative to control; SMG, smooth muscle genes; and TCF, ternary complex factor.

Recent work of Dubrac et al^31^ have shown that, under certain pathological conditions, pericytes can acquire disease-promoting properties. Using the oxygen-induced retinopathy (OIR) mouse model, it was shown that excessive PDGFB-PDGFRB signaling leads to pathological activation of pericytes, which promotes the formation of neovascular tufts (NVT). NVTs are clustered capillary loops, which show excessive EC proliferation and extravasation of red blood cells (RBCs). During OIR, pericytes undergo a pathological switch accompanied by upregulation of SMGs, which is characterized by strong expression of αSMA. Because our results suggested that SRF regulates both pericyte migration and expression of αSMA, we hypothesized that SRF might be a driving force of pathological pericyte activation during ischemic conditions. To address this hypothesis, we performed OIR experiments and kept P7 *Srf-flex1::Pdgfrb-CreER*^T2^ and control pups for 5 days under hyperoxic conditions, which led to vaso-obliteration in the primary vascular plexus (Figure 4A). We then returned the mice to normal oxygen conditions (21% O_2_), which provoked a strong hypoxic response and resulting pathological revascularization and NVT formation. During the revascularization phase, we applied tamoxifen to the pups (P12–P14) to induce *Srf^iMCKO^* and analyzed the impact on NVT development at P17.

Stainings with the endothelial marker CD31 revealed a significant reduction of NVT development by 38.5% (±9.5%; *P*=0.0025; Figure 4B and 4C) and exhibited an improved revascularization, evident as a reduction of the avascular area by 35.2% (±21.2%; *P* =0.00053) in *Srf^iMCKO^* OIR retinas. Costainings for NG2 and desmin confirmed the presence of pericytes on NVTs both in control and *Srf^iMCKO^* OIR retinas (Figure 4D; Figure S3A and S3B). Although in control retinas pericytes on NVTs displayed the characteristic upregulation of αSMA (Figure 4D), the αSMA staining in *Srf^iMCKO^* OIR retinas was reduced by 92% (Figure 4D and 4E). We further confirmed by quantitative PCR analysis that mRNA expression of *Acta2*, the gene encoding αSMA and other SMC genes was almost completely lost in *Srf^iMCKO^* OIR retinal lysates (Figure 4F; Figure S3D), which strongly suggests that pathological activation of pericytes was diminished. We did not observe a compensatory upregulation of the muscle genes *Actc1* or *Actg1* (Figure S3C). In contrast, mRNA levels of *Vegfa*, the main angiogenic driver, were significantly reduced (Figure 4F). This, in turn, could explain the reduction in NVT formation and decrease in avascular area.

Taken together, our results show that SRF is a necessary player in the pathogenic activation of pericytes during OIR and that αSMA upregulation, a common finding in several vascular conditions, relies on the activation of this transcription factor. The relevance of SRF for the phenotypic switch of pericytes during OIR makes it a potential therapeutic target to prevent pathological activation of pericytes although its role during physiological angiogenesis (Figure 4G) should not be overlooked.

### SRF Deletion in MCs Triggers the Formation of Arteriovenous Malformations

Since in *Srf^iMCKO^* mice, *Srf* is not only deleted in pericytes but also in vSMCs, we wanted to study its requirement for vSMC development and function in further detail. To visualize vSMCs during early retinal development, we performed immunohistochemical staining for αSMA and NG2 at P6. In control retinas, αSMA strongly highlighted part of the contractile machinery located in the cytoplasm of vSMCs, whereas membrane-bound NG2 staining outlined the cell shape. Remarkably, in vSMC of *Srf^iMCKO^* retinas, the αSMA signal was lost (Figure S4A). Quantitative PCR analysis of whole retina lysates from *Srf-KO* pBPCs confirmed a dramatic drop in *Acta2* gene expression (Figure S4B). Despite the complete absence of the αSMA signal, NG2 staining still marked the vSMC population on arteries (Figure S4A and S4B), indicating that vSMCs were present but had lost *Acta2* expression. An in-depth analysis of NG2-positive cell coverage on arteries indicated only a slight reduction of vSMCs in *Srf^iMCKO^* retinas (Figure S4B). These results strongly argued that SRF is strictly required for *Acta2* expression in arterial vSMCs.

**Figure 5.**
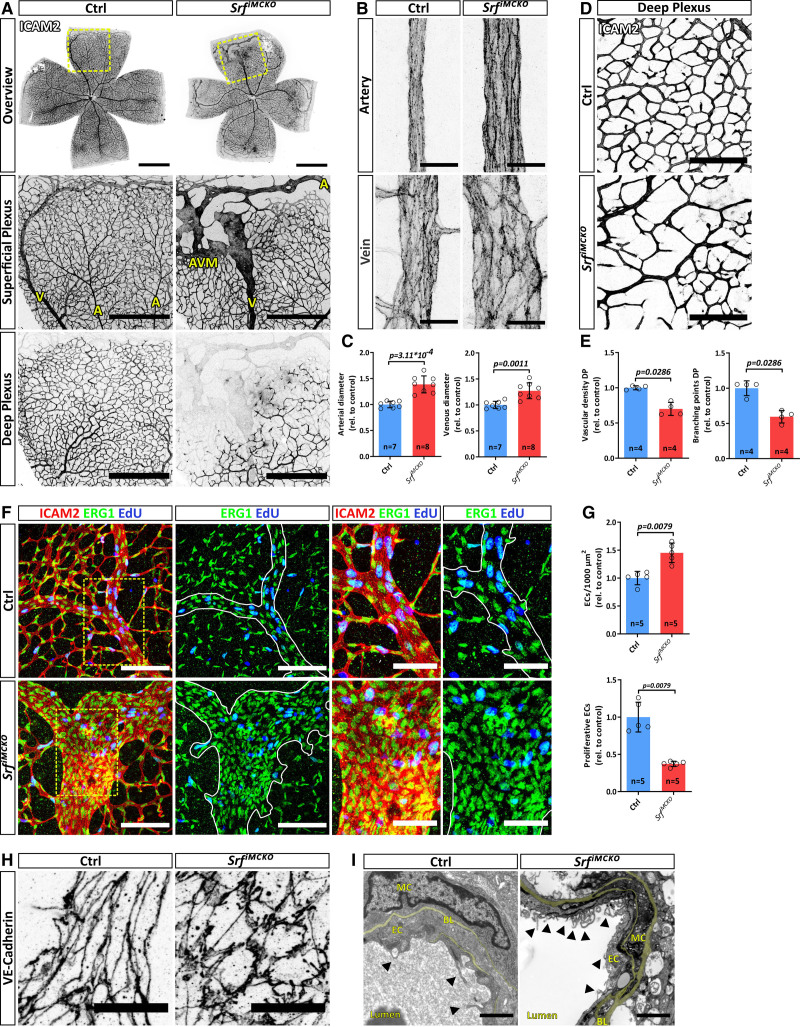
**SRF (serum response factor)-deficient vascular smooth muscle cells (vSMCs) trigger the formation of arteriovenous (AV) shunts. A**, Epifluorescence overview (**upper** row) and confocal images (**lower**) of P12 control (Ctrl) and *Srf^iMCKO^* retinal whole-mounts, stained using the endothelial marker ICAM2 (intercellular adhesion molecule 2). The yellow dashed squares indicate magnified regions of superficial and deep plexus (**middle** and **lower** columns). Arteries (A), veins (V), and the AV malformation (AVM) are annotated in yellow letters. Scale bar: 1 mm (**upper** row), 500 µm (**middle** and **lower** row)**. B**, Confocal, images of representative arteries and veins indicating the dilation of both vessel types in *Srf^iMCKO^* retinas. Scale bar, 25 µm. **C**, Quantification of artery and vein diameters. **D**, Representative confocal image of the deep plexus. Scale bar, 150 µm. **E**, Quantification of the vascular density. **F**, AV shunts and control veins, showing proliferating cells (EdU [5-ethynyl-2′-deoxyuridine], blue), blood vessels (ICAM2, red), and endothelial nuclei (ERG1 [early growth response 1], green). White lines define the border of malformations or respective control positions and outline the vessel shape (second and fourth column). Images in the third and fourth columns show magnification of the yellow dashed squares of the parts in the first column. Scale bar, 100 µm (**left**) and 50 µm (**right**). **G**, Quantification of the endothelial cell (EC) density (ERG1^+^ counts/ICAM2+ area) and EC proliferation (ERG1^+^+EdU^+^/ERG1^+^ counts). **H**, High-resolution confocal image of the junctional EC-specific protein VE-cadherin (vascular endothelial cadherin), visualizing the shape of endothelial cells. Scale bar, 20 µm. **I**, Electron microscopy images of control veins and malformed *Srf^iMCKO^* veins visualizing ultrastructural changes. Black arrowheads pointing to EC membrane invaginations. Note also that the basal lamina (BL, pseudocolored in yellow) is thickened in *Srf^iMCKO^*. Scale bar, 1 µm. Error bars indicate SD of the mean. Statistical significance in **C** was determined using the Shapiro-Wilk test for normality followed by an unpaired *t* test with Welch correction (2-tailed). For the data shown in **E** and **G**, the unpaired Mann-Whitney (2-tailed) was used. Number of analyzed animals (n) is indicated. DP indicates deep plexus; MC, mural cell; and rel., values relative to control.

To investigate the impact of *Srf* deletion during vascular remodeling, we analyzed retinas at P12. At this stage, blood vessels sprout perpendicular from the primary retinal plexus to form the deep plexus.^32^ The tissue undergoes extensive remodeling and both arteries and veins progressively mature. vSMCs become increasingly important as the blood pressure in arteries increases, and consequently, contractile functions are crucial to regulate the vascular tone. At this stage, control retinas displayed a stereotypical vascular pattern, with hierarchical organized blood vessels and a clear arterial-venous zonation, implicating that blood flow is channeled from arteries into arterioles, capillaries, venules, and subsequently into veins. In contrast, we observed severe patterning defects in *Srf^iMCKO^* retinas (Figure 5A), in which both arteries and veins were significantly dilated (Figure 5B and 5C). Moreover, arteries often were directly connected to veins, a phenomenon called arteriovenous shunting (Figure 5A). The shunting phenotype was highly penetrant since we observed at least one arteriovenous shunt in every analyzed P12 retina (Figure S5B). In addition, we noticed that the deep vascular plexus was considerably under-developed, as illustrated by a reduced vascular area and a decreased number of vessel branch points (Figure 5D and 5E). Remarkably, deep plexus capillaries were completely absent directly underneath the arteriovenous shunts (Figure 5A).

To clarify if arteriovenous identity is lost in association with shunt formation, we performed immunohistological staining with known markers of arteriovenous-zonation on control and *Srf^iMCKO^* retinas at P12 (Figure S4C and S4D). For this purpose, we used SOX17 (SRY-box transcription factor 17), an arterial marker necessary for acquisition and maintenance of arterial identity,^33^ and Endomucin, a transmembrane sialomucin expressed solely on the surface of capillary and venous, but not arterial, endothelium.^34–36^ As expected, control retinas displayed a clear arteriovenous zonation with arteries strongly positive for Sox17 and negative for Endomucin, whereas veins were strongly positive for Endomucin, but showed only a weak signal for Sox17 (Figure S4D). This pattern of arteriovenous-zonation was maintained in *Srf^iMCKO^* retinas, arguing that the vascular malformations are indeed arteriovenous shunts and not dilated, dedifferentiated vessels. We further noticed that, in *Srf^iMCKO^* retinas, AVMs became more pronounced over time due to a constant increase in artery and vein diameter and concomitant loss of intermediate capillary plexus (Figure S5A). Although at P12 some malformations still contained capillary remnants between arteries and veins, the number of AVMs in which arteries and veins directly connected more than doubled at P25, whereas the total amounts of AVMs remain unchanged (Figure S5B and S5C).

The arteriovenous shunts in *Srf^iMCKO^* retinas morphologically resembled vascular malformations characteristic of mouse models of hereditary hemorrhagic telangiectasia (HHT), a disease caused by mutations in the TGF (transforming growth factor)-β pathway.^37^ In HHT mouse models, it has been reported that ECs that contribute to shunt formation proliferate at higher rates than neighboring ECs.^38,39^ To clarify if a similar mechanism could explain arteriovenous shunt formation in *Srf^iMCKO^* retinas, we performed in vivo proliferation assay using EdU in combination with the nuclear EC marker ERG1. Whereas ERG1 staining revealed that EC density was locally increased in the malformed areas, EC proliferation (ERG1^+^ EdU/ERG1^+^ counts) was markedly reduced in *Srf^iMCKO^* retinas (Figure 5F and 5G). Immunostainings with the junctional marker VE-cadherin (vascular endothelial cadherin) further demonstrated that the shape of EC in the malformed regions was severely affected. Although ECs on veins in control retinas were elongated and showed a spindle-like morphology with straight adherens junctions, ECs at arteriovenous shunts had a round morphology, were less elongated, and their adherens junctions appeared irregular with partially overlapping areas and a zig-zag morphology (Figure 5H). Furthermore, transmission electron microscopy of arteriovenous shunt regions revealed enlarged basal laminae and intraluminal membrane invaginations originating from ECs (Figure 5I). Interestingly, intraluminal membrane invaginations have also been described in *Pdgfb* mutant mice, in which pericyte recruitment is defective.^40^

### SRF Is Critical for the Expression of Contractile Genes in vSMCs

**Figure 6.**
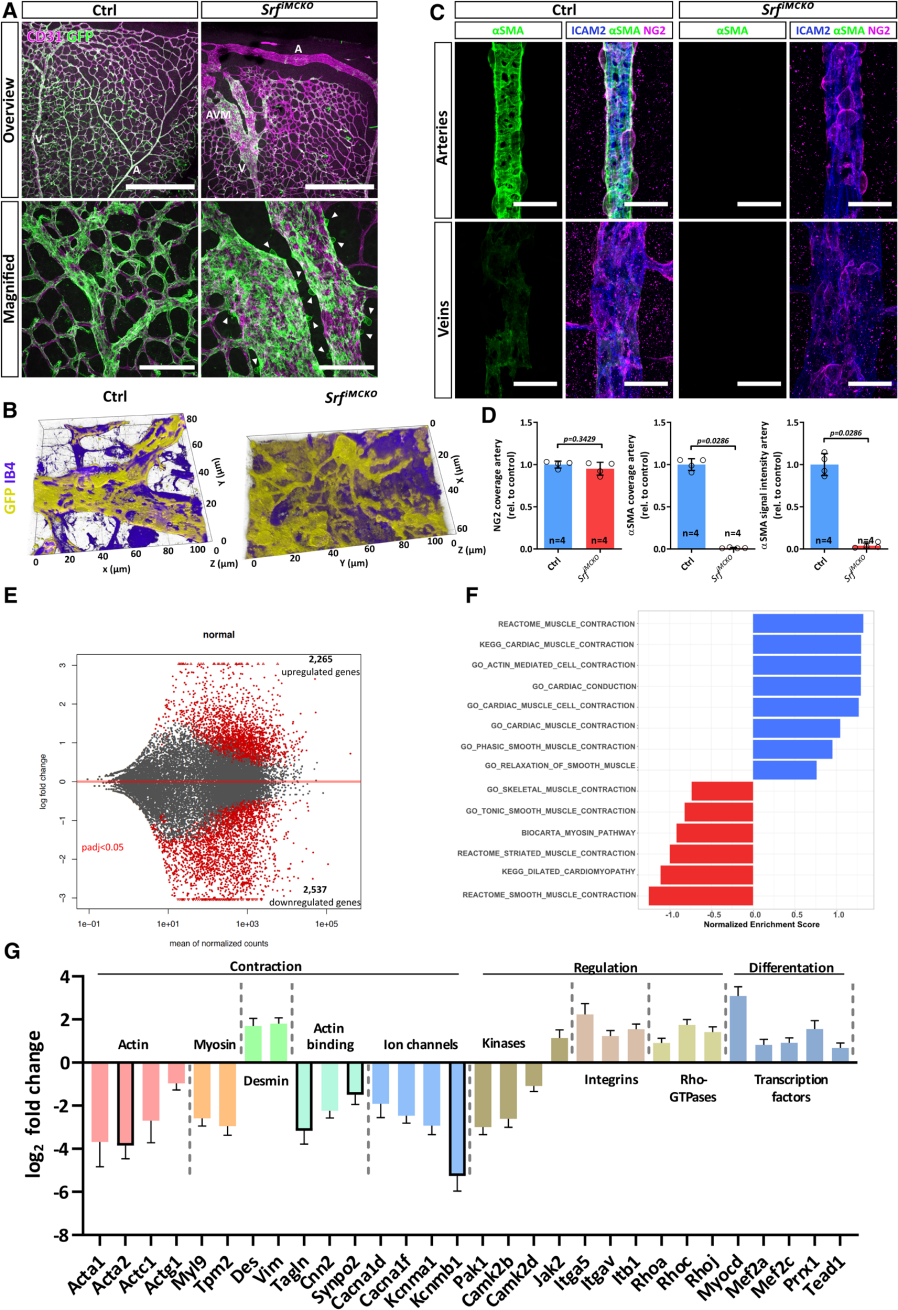
**Dysregulated expression of contractile genes in SRF (serum response factor)^iMCKO^ mural cells (MCs). A**, Confocal images of retinal vasculatures genetically labeled with the *Rosa26^mTmG^* reporter for MCs (GFP, green) and costained for CD31 (endothelial cells [ECs], blue). Arteries (A), veins (V), and arteriovenous malformation (AVM) are highlighted with white letters. Note MC-free area of arteries at the periphery in the *Srf^iMCKO^* retina. White arrowheads point at MCs, which round up and appear to detach from the endothelium in Srf^iMCKO^ animals. Scale bars, 500 µm (**top**) 100 µm (**lower**). **B**, Three-dimensional (3D) rendering of confocal images showing morphological abnormalities of MCs in arteriovenous (AV) shunts of *Srf^iMCKO^* retinas and corresponding venous areas in control (Ctrl) retinas. **C**, Confocal images of arteries and veins of *Srf^iMCKO^* and respective control retinas stained for αSMA (alpha-smooth muscle actin; green), ICAM2 (intercellular adhesion molecule 2; blue), and NG2 (neural/glial antigen 2; magenta). Note the loss of αSMA signal in arterial and venous MCs of *Srf^iMCKO^* retinas despite the presence of NG2. Scale bar, 25 µm. **D**, Quantification of aSMC coverage (NG2- or αSMA-positive area normalized to ICAM2 positive area) and αSMA signal intensity on arteries. Error bars show SEM. Statistical comparison by the unpaired Mann-Whitney test (2-tailed). Number of analyzed animals (n) is indicated. **E**, Volcano plot displaying differentially expressed genes from RNA-sequencing (RNA-Seq) analysis. Red dots indicate significantly (*P*<0.05) dysregulated genes. DeSeq2 package was used for differential gene expression analysis across samples for protein-coding genes. **F**, Gene set enrichment analysis (GSEA) of RNA-Seq dataset of *Srf^iMCKO^* and control MCs. Positive normalized enrichment scores (NES) indicate pathways containing downregulated genes whereas negative NES indicate pathways containing genes that are upregulated. Representative summary of tested contraction-related gene sets within the Gene Ontology (GO), Reactome, and Biocarta databases. **G**, Gene expression log2 fold changes of selected genes identified by RNA-Seq and subsequent GSEA. Listed genes are essential contributors to smooth muscle cell (SMC) contraction and were functionally grouped (headings). Framed bars indicate frequently used SMC markers. Error bars indicate the SEM. All presented genes were significantly dysregulated (*P*<0.05). GFP indicates green fluorescent protein; IB4, isolectin B4; KEGG, Kyoto Encyclopedia of Genes and Genomes; and rel., values relative to control.

Although, overall, we only observed a marginal difference in MC coverage between control and *Srf^iMCKO^* retinas (Figure 6A; Figure S6A through S6D), we noticed, in arteriovenous shunt areas, that the venous shunt portions showed an increased vSMC coverage, whereas arterial shunt portions were often deprived of vSMCs (Figure 6A; Figure S6C and S6D). A 3-dimensional segmentation analyses of arteriovenous shunt regions showed striking morphological alterations of vSMCs (Figure 6B) and high-resolution imaging further revealed changes in the intracellular organization of the cytoskeletal protein Desmin (Figure S6E). Taken together with the complete absence of αSMA (Figure 6C and 6D) these results suggested that the contractile ability of vSMCs is compromised in *Srf^iMCKO^* retinas. To further investigate a potential reduction of vSMC contractility, we determined the expression levels of genes, encoding typical contractile proteins in SMCs^29,30^ and found that *Acta2*, *Mhy11*, and *Tagln* were strongly downregulated in whole retinal lysates of *Srf^iMCKO^* mice (Figure S7A). We further used pBPCs cultures to study the expression of contractile genes. In control pBPCs cultures, the addition of serum leads to a strong induction of SMGs, such as *Acta2*, *Mhy11*, and *Tagln*, whereas in *Srf-KO* cells the expression of those genes was almost completely lost in any tested condition (Figure S7B). Altogether, these results show that SRF is indispensable for the expression of genes responsible for the contractile abilities of vSMCs.

To characterize the transcriptional changes that result from SRF deletion in MC in further detail, we utilized an RNA-sequencing approach. We induced control and *Srf^iMCKO^* mice with tamoxifen from P1 to P3 and FAC-sorted PDGFRB^+^ MCs from retinas at P12. We subsequently isolated RNA from those cells and sequenced in triplicates. Expression analysis confirmed a high enrichment of mural-specific genes, such as *Pdgfrb*, *Rgs5*, and *Notch3*, whereas genes typically expressed in other cell types, such as ECs (*Pecam1*, *Cdh5*) or astrocytes (*Gfap*), were underrepresented, which suggested that sufficiently pure MC fractions had been isolated and sequenced (Figure S7C). Principal component analysis of all sequenced datasets showed a sufficient reproducibility between samples within one group and a strong difference between the control and the *Srf^iMCKO^* group (Figure S7D).

Differential gene expression analysis using a false discovery rate–adjusted *P* <0.05 and an absolute log_2_ fold change >0.5 identified 2265 upregulated and 2537 downregulated genes (Figure 6E). Notably, we identified 517 differential expressed genes (350 up/167 down) which are potentially under control of SRF.^41,42^ The expression of the majority of these differentially expressed genes (375) is controlled by the MRTF-SRF signaling axis (Figure S7E). Further, gene set enrichment analysis using the Kyoto Encyclopedia of Genes and Genomes and the Gene Ontology databank resulted especially in the identification of pathways linked to contractile functions of myocytes and SMCs (Figure 6F). Importantly, the gene set enrichment analysis identified numerous differential expressed genes crucial for smooth muscle contraction (Figure 6G), and the downregulation of *Acta2* and *Tagln* in *Srf^iMCKO^* MCs could be confirmed. Strikingly, we also observed a strong downregulation of numerous structural elements of the contractile apparatus, such as myosin light chain 9 (*Myl9*; log_2_fc: −2.59±0.36), tropomyosin beta chain (Tpm2; log_2_fc: −2.96±0.42), and several members of the actin family, such as *Acta1* (log_2_fc: −3.68±1.14), *Actc1* (log_2_fc: −2.69±1.03), and *Actg1* (log_2_fc: −0.97±0.31). Of note, multiple ion channel proteins important for Ca^2+^ release into the cytoplasm, which triggers vSMC contraction, were markedly downregulated (*Cacna1d*, log_2_fc: −1.91±0.64; *Cacna1f*, log_2_fc: −2.46±0.34; *Kcnma1*, log_2_fc: −2.92±0.42 and *Kcnmb1*, log_2_fc: −5.27±0.7). Furthermore, we noticed a partial downregulation of kinases which are involved in the regulation of the vascular tone (Camk2b [calcium/calmodulin-dependent protein kinase 2]; log_2_fc: −2.61±0.39 and Camk2d 1.09±0.26 and Pak1 [p21 activated kinase 1]; log_2_fc: −3.0±0.34). Conversely, several integrins, Rho-GTPases, and transcriptional elements were found to be upregulated in *Srf^iMCKO^* MCs. Most notably the SRF-cofactor myocardin (*Myocd*, log_2_fc: 3.08±0.43), which binds to and activates SRF and thereby leads to induction of muscle-specific gene expression.^43^ The upregulation of those genes might reflect a compensatory mechanism aimed to limit the consequences that the loss of SRF caused for MC function.

Previous studies have shown that under pathological conditions KLF4 (krueppel-like factor 4) activates promotes a phenotypic switch of SMCs from a contractile phenotype towards a mesenchymal phenotype (reviewed by Yap et al^44^ 2021). To investigate if in *Srf^iMCKO^* mice MCs shift towards a mesenchymal phenotype, we analyzed our RNA-seq data for the expression of relevant marker genes. We found that the expression of the mesenchymal markers *Ly6a* and *Cd34*, commonly used to identify such transition^45,46^ were not increased (Figure S7F). Immunostaining for the corresponding proteins SCA1 (stem cells antigen-1) and CD34 only showed detectable labeling in ECs (Figure S7G and S7H), indicating that the expression of these markers in retinal vSMC is generally very low. In addition, changes in KLF4 expression were not significant and expression levels of *Atf4*, a transcription factor known to prevent proteasomal degradation of KLF4 and to promote the phenotypic switch of SMC towards a fibroblast or macrophage-like phenotype^45^ was reduced (Figure S7F). The expression levels of *Lum* and *Dcn*, markers that would indicate a switch towards a fibroblast-like phenotype,^47^ were very low in both control and *Srf^iMCKO^* conditions and nonsignificantly changed (Figure S7F). Taken together, our data do not indicate a switch of *Srf^iMCKO^* SMCs towards a mesenchymal or fibroblast-like phenotype.

### Loss of Vascular Tone in the Absence of Mural SRF

**Figure 7.**
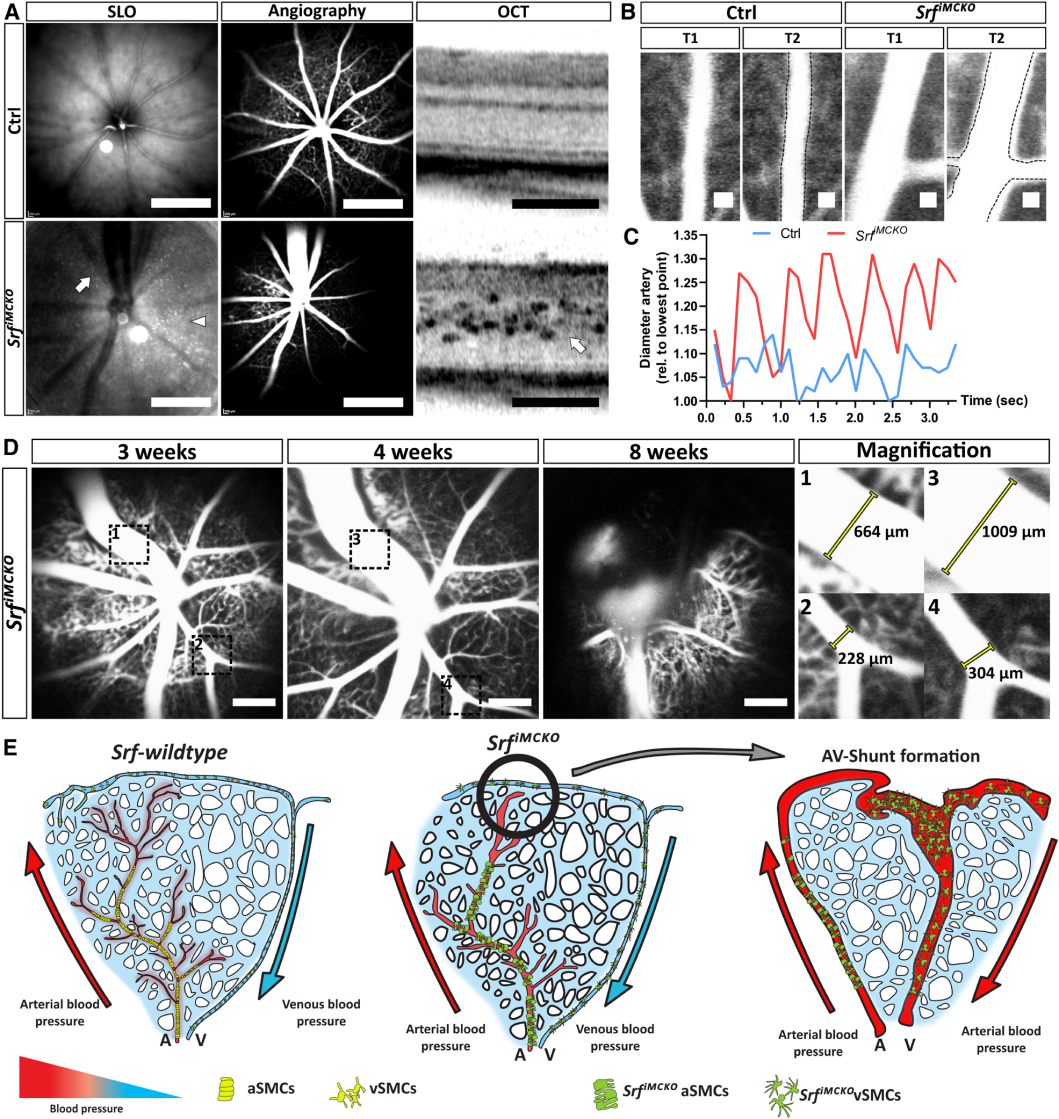
**Srf^iMCKO^ vascular smooth muscle cells (vSMCs) lose their ability to maintain the vascular tone. A**, In vivo, live imaging of 4 weeks old control and *Srf^iMCKO^* mouse retinas by scanning laser ophthalmoscopy (SLO) and optical coherence tomography (OCT). Native SLO and indocyanine green perfused angiography highlights vessel structure and perfusion, whereas OCT imaging highlights optical section of the retina. Scale bars, 2 mm (SLO and angiography), 200 µm (OCT). **B**, Representative control (Ctrl) and Srf^iMCKO^ artery at two time points (T1 and T2) indicating vessel movements. The dashed black line indicates T1. Scale bar, 100 µm. **C**, Respective measurement of arterial vessel diameter shown in **B**. **D**, Serial angiography imaging of the same *Srf^iMCKO^* eye at 3, 4, and 8 weeks. Note the rupture of the venous vessel at 8 weeks. The **right** shows magnification of the dashed black boxes numbered in the first 2 parts. Venous (1 and 3) and arterial (2 and 4) diameters are annotated. Scale bar 1 mm. **E**, Model depicting how the loss of vascular tone could trigger the development of arteriovenous (AV) shunt formation in *Srf^iMCKO^* mutant mice. A indicates arteries; aSMCs, arterial smooth muscle cells; V, veins; and vSMCs, venous smooth muscle cells.

Our transcriptomic analyses of MCs, isolated from P12 *Srf^iMCKO^* and control retinas revealed a conspicuous misregulation of genes involved in the regulation of the vascular tone. To explore the biological significance of these results, we investigated the retinal vascular morphology of control and *Srf^iMCKO^* mice at 3, 4, and 8 weeks of age in vivo via scanning laser ophthalmoscopy and optical coherence tomography. Scanning laser ophthalmoscopy confirmed a substantial enlargement of arterial and venous vessels in all examined *Srf^iMCKO^* retinas (Figure 7A). Moreover, we were able to classify the individual vascular alterations into three different degrees of severity, ranging from mild and intermediate to severe (Figure S8A and S8B). Strikingly, there were also major abnormalities in the dynamic movement of vessel walls associated with blood pulsations in *Srf^iMCKO^* retinas. Although the vessel wall of arteries in control retinas showed relatively scant movements, we observed exceptionally strong pulsating motion in the arterial wall of *Srf^iMCKO^* animals (Figure 7A through 7C; Video S4). This finding is in strong support of a hemodynamically relevant loss of the vascular tone. Moreover, in our longitudinal study, we further observed that arterial and venous vessels gradually enlarged during the course of the experiment. This effect was quantified by repetitive size assessment of identical vessels at 3, 4, and 8 weeks, respectively (Figure 7D). In the most severe cases, we observed that venous vessels ruptured (Figure 7D), which further supports the hypothesis that vSMCs had lost their ability to modulate blood pressure.

To investigate potential perfusion problems that could result from a loss of vascular tone, we analyzed *Srf^iMCKO^* and control retinas with scanning laser ophthalmoscopy angiography, for which we used indocyanine green as a contrast agent. These results revealed profound alterations of the capillaries in *Srf^iMCKO^* retinas, which are suggestive of associated perfusion defects (Figure 7A; Figure S8A and S8B).

### Redirected Blood Flow via Arteriovenous Shunts Leads to Reduced Capillary Perfusion

**Figure 8.**
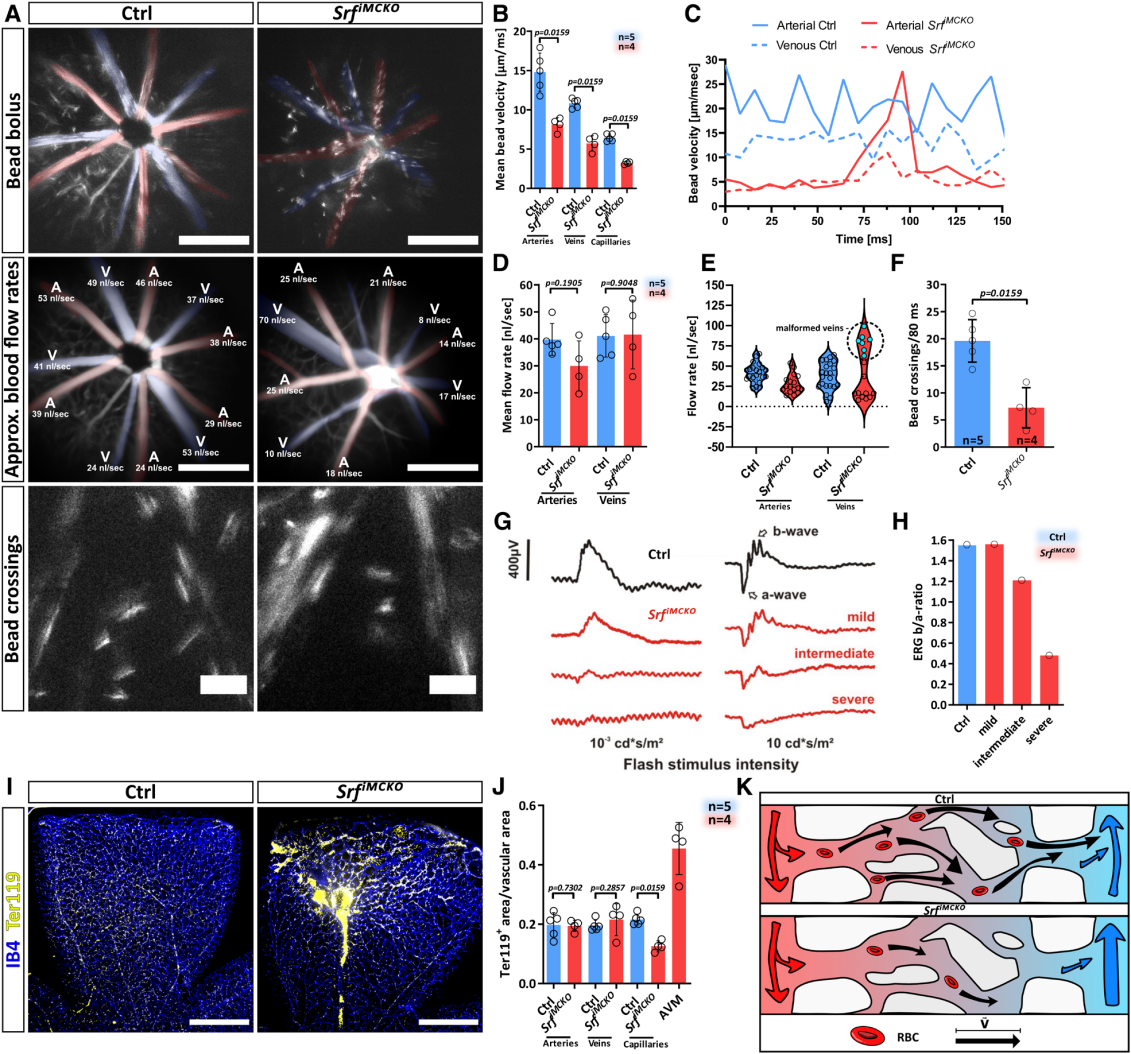
**Redirection of blood flow in arteriovenous (AV) shunts leads to reduced capillary perfusion. A**, Intravital imaging of retinal blood flow in control (Ctrl) and *Srf^iMCKO^* retinas. Images in the first row show a bolus injection of 2 µm fluorescent beads. Note that the length of bead strokes indicate bead velocity. Scale bar 500 µm. Parts in the second row show representative images of cadaverine perfusion and calculation of flow rates (nL/s). Scale bar 500 µm. Parts in the third row show a snapshot of fluorescent beads, crossing through capillaries between arteries and veins. Scale bar 100 µm. Arteries (A; red) and veins (V; blue) are pseudocolored in the first two rows (**B**) Quantification of mean bead velocity in arteries, veins, and capillaries in control and *Srf^iMCKO^* retinas (**C**) Representative measurement of bead velocity over time in arteries and veins. **D**, Quantification of mean blood flow rates. **E**, Distribution of flow rates in individual arteries and veins of control and *Srf^iMCKO^* retinas. Note that flow rates in malformed veins (encircled) are elevated compared to nonmalformed veins. **F**, Quantification of bead crossing between arteries and veins via capillaries (**G**) Representative examples of in vivo electroretinography (ERG) data from the scotopic single flash intensity series, obtained from a control and 3 differently affected mutant animals at the age of 4 weeks. Note the reduced overall size of responses in the mutants, together with a relatively strong reduction of the b-wave component in comparison to the a-wave component. **H**, Quantification of the ERG b/a-ratio for the results shown in (E) indicating increased retinal hypoxia and a functional deficit of vision (**I**) image of P12 control and *Srf^iMCKO^* retinal whole-mounts, stained for the endothelial marker isolectin B4 and the erythrocyte marker Ter119. Note that the majority of erythrocytes are localized in malformed vessels. **J**, Quantification of Ter119^+^ cell distribution in different vessel types. Scale bar 500 µm. **K**, Schematic representation of reduced capillary perfusion in *Srf^iMCKO^* retinas as a result of redirected blood flow via AV shunts. Error bars show SEM. Statistical analysis by the unpaired Mann-Whitney test (two-tailed). Number of analyzed animals (n) is indicated. RBC indicates red blood cells; and SRF, serum response factor.

We next performed intravital imaging of the retinal vasculature in *Srf^iMCKO^* and control animals to analyze if changes in capillary perfusion are a consequence of a pathological blood flow redirection via the observed arteriovenous shunts. To do so, fluorescent microspheres (beads) were injected into the circulation and imaged to calculate flow velocity and distribution over time. Bead velocity and distribution has previously been established to be comparable to labeled RBCs and allows to determine RBC velocity and approximate blood flow rates (nl/s)^48^ (Figure 8A through S8F; Figure S8E; Video S5 and S6). Overall, we observed a substantial reduced mean velocity of RBCs in *Srf^iMCKO^* vasculature. This is likely a direct consequence of the lack in myogenic response (Figure 8A through 8C; Figure S8E; Video S5). In contrast, the mean arteriovenous-blood flow rate was not significantly changed in *Srf^iMCKO^* retinas (Figure 8D and 8E). However, when comparing flow rates of individual vessels, it became apparent, that *Srf^iMCKO^* veins experience huge variations in flow rates and that the blood flow is predominantly redirected through shunting veins, whereas nonshunting veins experience lower flow rates (Figure 8A and 8E). As a result, capillary perfusion was also significantly reduced in *Srf^iMCKO^* retinas. This was apparent by a reduced number of beads crossing through capillaries between adjacent arteries and veins (Figure 8A and 8F; Video S6). A postmortem analysis by immunolabeling of RBCs by Ter119 in the retinal vasculature of P12 animals confirmed these observations and demonstrated that RBCs accumulate in AVMs, whereas their presence is reduced in the capillary network (Figure 8I through 8K).

The reduced capillary perfusion likely has detrimental effects on the retinal tissue, as oxygenation and nutrient supply are expected to be severely decreased. Consequently, we performed an assessment of retinal function via electroretinography. The electroretinogram is a measure of retinal sum potentials triggered by a brief light stimulus and mainly comprises the transient activity of photoreceptors and bipolar cells. A series of light flashes of increasing intensity is commonly employed to investigate the integrity of the visual system at the level of mainly the outer retina.^49^ A typical finding in the dark-adapted (scotopic) electroretinography in case of retinal hypoxia is a discrepancy between the initial negative wave (the a-wave) and the subsequent positive wave (the b-wave), leading to a waveform shape called negative electroretinography. Indeed, we clearly observed negative scotopic electroretinography in eyes of *Srf^iMCKO^* animals as well as reduced b/a-wave ratios (Figure 8G and 8H), indicating reduced retinal oxygenation, most likely as a result of defective capillary perfusion. The grade of severity of vascular alterations was well correlated with the scotopic electroretinography measurements (Figure 8G and 8H).

To analyze if SRF is also needed for MC function in the mature vasculature, we induced its deletion in adult mice. We injected 8-week-old *Srf-flex1::Pdgfrb-CreER*^T2^ and littermate control mice with tamoxifen on 5 consecutive days (Figure S9A) and analyzed retinas after 2 and 12 months respectively. After 2 months, the retinas showed no obvious vascular phenotype (data not shown). However, after 1 year, we observed a significant dilation of arteries and veins (Figure S9B and S9C). Coimmunostainings for Desmin and αSMA did not indicate a substantial loss of vSMC coverage on arteries or veins but revealed a marked reduction of αSMA expression, especially on veins (Figure S9D and S9E). This suggests that SRF deletion in adult mice leads to a reduction in contractile protein expression rather than a loss of vSMCs. In support of this hypothesis, we also observed a reduced expression of *Acta2* and *Tagln* in whole brain lysates of in *Srf^iMCKO^* mice (Figure S9F). In contrast, we did not observe changes in the capillary network of those mice and conclude that SRF is dispensable in adult retinal pericytes.

## Discussion

Our understanding of MC function has increased considerably in the past decade. It is now well established that pericytes play a key role in maintaining the integrity of the blood-brain barrier and that their dysfunction contributes to the progression of numerous diseases (reviewed in Geranmayeh et al,^50^ Hirunpattarasilp et al,^51^ and Brown et al^52^). Yet, despite recent advances, many aspects of pericyte biology still remain poorly understood.

During angiogenesis, pericytes are recruited to sprouting blood vessels via PDGFB/PDGFRB signaling.^11,53^ PDGFB, which is produced and secreted by tip cells is retained in the ECM (extracellular matrix) of new vessel sprouts via its retention motif.^54^ Pericytes, which in turn express PDGFRB, sense the PDGFB tissue gradient and comigrate along the nascent vessel sprouts.^55^ NCK1 (non-catalytic region of tyrosine 1) and NCK2 adaptor proteins have been proposed to mediate PDGFB-dependent PDGFRB phosphorylation.^31^ However, which signaling molecules are activated downstream of PDGFRB during pericyte recruitment and how those molecules regulate the cytoskeleton to mediate cell motility has not been well characterized. Here, we demonstrate that PDGFB/PDGFRB signaling triggers translocation of MRTF coactivators to the nucleus, where they associate with the SRF transcription factor and activate expression of a specific gene set that subsequently regulates pericyte migration. Interestingly, MRTF-driven activation of SRF has previously been reported in response to PDGFRα signaling in mesenchymal cells during craniofacial development,^56^ suggesting that MRTF/SRF activation might be a conserved feature downstream of PDGFRs. Our data suggest that SRF is a key regulator of cytoskeletal functions in pericytes, as its deletion (*Srf^iMCKO^*) led to severe cytoskeletal and migratory defects in vivo. As a consequence, SRF-depleted pericytes were unable to fully populate the retinal vasculature, which resulted in a reduced pericyte coverage, especially at the sprouting front, and caused vessel dilation as well as reduced barrier properties.

Recent studies have highlighted that pericytes are not only crucial for normal vascular development and for maintaining blood-brain barrier properties in the adult vasculature but can also acquire disease-promoting properties. Examples are the formation of vascular malformations as a consequence of RBP-J (recombination signal binding protein for immunoglobulin kappa J region) deletion in pericytes and their role as promoters of NVT formation in ischemic retinopathy.^31,57^

Here, we demonstrate that the pathological features of pericyte activation in ischemic retinopathy are mediated by SRF, which regulates pericyte migration downstream of PDGFRB signaling and activates the expression of SMGs. Accordingly, OIR experiments in *Srf^iMCKO^* mice showed reduced NVT formation and improved revascularization. Remarkably, the pathological αSMA expression in pericytes was completely prevented.

In this regard, it is interesting to note that pathological activation of pericytes shares certain similarities with fibrotic reactions in which SMGs are expressed at high levels and excessive amounts of ECM proteins are deposited.^58^ The fibrotic reaction is regulated, at least in part by the actin-MRTF-SRF axis,^59^ and recently developed small molecule inhibitors that target MRTF function are promising candidates for the treatment of fibrosis.^60^

Besides its crucial role in pericytes, SRF also plays essential role in vSMCs, where it regulates the expression of SMGs. These genes typically contain a CArG (−cis) element that serves as an SRF binding side^41,61^ and, in part, encoded proteins that enable vSMCs to constrict and thereby increase vascular resistance.^62,63^ Through the modulation of vascular resistance, vSMCs can regulate blood flow to satisfy the local demands for oxygen and nutrients.^64^ This implies, that a vessel branch that experiences a stochastic increase in flow compared to its neighboring branch must be able to counterbalance that increased flow rate to ensure an equal blood distribution. This is attained by an increase in resistance in the affected branch due to vasoconstriction, which naturally leads to an increased flow in the neighboring branches, where resistance is lower.^65,66^ This property of vSMCs has been termed the myogenic response.^67^ In *Srf^iMCKO^* mice, vSMCs fail to express typical SMGs and are no longer able to mediate the myogenic response. Consequently, flow redistribution cannot be achieved and initial stochastic changes in local blood flow cannot be adequately redirected. We propose, that, as a consequence, some branches develop into arteriovenous shunts that funnel a proportion of the blood directly to the venous circulation (Figure 7E). This relives the pressure from surrounding vessels and ensures a certain functionality of the retinal vasculature. These shunts form, where one would expect, in the retinal periphery, where the distance between arteries and veins is the shortest. Intravital imaging of those AVMs revealed a pathological blood flow redirection primarily via shunting veins. As a consequence, capillary perfusion was significantly reduced in *Srf^iMCKO^* retinas. Electrophysiological measurements confirmed that retinal function and thus, vision, was severely impaired. In this context, it is interesting to note that similar roles of SRF have been reported in visceral SMCs, where *Srf* deletion led to impaired contraction and thus to severe dilation of the intestinal tract.^19,22^

The fully developed retinal vasculature seems to be more robust to changes. Mural *Srf* deletion in adult mice did not lead to arteriovenous shunt formation, which is likely attributed to the low plasticity of fully matured blood vessels. In this context, it is worth noting that adult deletion of SRF did not result in a complete loss of αSMA in arterial SMCs. It is thus possible, that the remaining αSMA protein levels maintain a sufficient degree of contractile function and that because of this, arteriovenous shunts did not form. However, the diameter of arteries and veins significantly increased in aged *Srf^iMCKO^* mice, and we observed a significant reduction of contractile proteins. In contrast, pericytes around capillaries seemed unaffected, suggesting that SRF is dispensable for pericyte homeostasis.

The finding that defective MC function can trigger the formation on AVMs might be of broader medical relevance. AVMs are hallmarks of HHT, a human disease caused by autosomal dominant mutations in genes of the TGF-β signaling pathway, in particular endoglin or ACVRL1 (activin receptor-like kinase 1).^37^ In HHT, AVMs commonly form in the nose, lungs, brain, or the liver and affected individuals often suffer from nasal and gastrointestinal bleedings. Rarely occurring AVMs in the central nervous system can even be life threatening.^68^ Thus far, MCs have not been directly implicated to trigger HTT-like malformations, although they have been found to be immature on AVMs and are thought to contribute to the instability of vessels.^69^ In addition, recent reports indicate the potential importance of MCs coverage in treatment of HHT.^69,70^ Furthermore, Sugden et al^71^ recently also highlighted the importance of hemodynamic forces in this context and demonstrated that endoglin function is necessary to mediate blood flow–induced EC shape changes which limit vessel diameter and prevent the formation of arteriovenous shunts. This is in line with our hypothesis, which suggests that blood vessel dilation and arteriovenous shunt formation can be triggered if hemodynamic changes are not counteracted. We propose, that, in *Srf^iMCKO^* mice, this is likely caused by the loss of the myogenic response. Our study suggests that vSMC can play a fundamental role in the development of AVMs and might put vSMC in AVM research a future focus.

## Article Information

### Acknowledgments

We thank Catrin Bitter and Lars Muhl for critically reading the manuscript. Furthermore, we thank Hyun-Woo Jeong and Caner Bagci for their help with the bioinformatic analysis. We are grateful to Anke Biedermann, Siegfried Alberti, and the staff of the University Clinic Tuebingen (UKT) animal facility for support with mouse husbandry. We want to thank Christian Feldhaus (light microscopy facility, MPI Tuebingen), Kristin Bieber (fluorescence-activated cell sorting [FACS] facility, UKT), Gabi Frommer-Kästle (electron microscopy facility, UKT), the BioVis facility Uppsala, Hans Schoofs, Mark Richards, Henrik Ortsäter and Thommie Karlsson for technical support.

### Sources of Funding

This study was supported by the Deutsche Forschungsgemeinschaft (DFG, German Research Foundation) Sonderforschungsbereich (SFB)/Transregional (TRR) 209 (Project number 314905040), the Karl-Kuhn foundation and the IMPRS Tuebingen “From Molecules to Organisms” to A. Nordheim and M.M. Orlich. The DFG (R.H. Adams: FOR 2325 and project number 391580220),the DFG (M.M. Orlich: 503902844), the Gustaf Adolf Johansson’s foundation (M.M. Orlich: 890622-p133), the European Research Council (C. Betsholtz: AdG294556), the Leducq Foundation (C. Betsholtz: 14CVD02; Ralf Adams: 18CVD03), the Swedish Cancer Society (C. Betsholtz: 150735), Knut and Alice Wallenberg Foundation (C. Betsholtz: 2015.0030) and (2020.0057 C. Betsholtz and K. Gaengel), Innovative Medicines Initiative (C. Betsholtz: IM2PACT-807015), the Swedish Research Council (C. Betsholtz: 2015-00550; K. Gaengel: 2021-04896) the Tore Nilsons Stiftelse för Medicinsk Forskning (K. Gaengel: 2020-00873).

### Disclosures

None.

### Supplemental Materials

Supplemental Methods

Figures S1–S9

Videos S1–S6

References 72–76

## Supplementary Material


