# Supplementary material for: Mural Cell SRF Controls Pericyte Migration, Vessel Patterning and Blood Flow
Source: Circ Res. 2022 Jul 14;131(4):308–27. doi: 10.1161/CIRCRESAHA.122.321109 (PMC9348820; doi:10.1161/CIRCRESAHA.122.321109)
Supplement: Supplementary file 1 [file res-131-308-s001.pdf]

Major Resources Table

In order to allow validation and replication of experiments, all essential research materials listed in the Methods should be included in the Major Resources Table below. Authors are encouraged to use public repositories for protocols, data, code, and other materials and provide persistent identifiers and/or links to repositories when available. Authors may add or delete rows as needed.

Animals (in vivo studies)

Species	Vendor or Source	Background Strain	Sex	Persistent ID / URL

Genetically Modified Animals

	Species	Vendor or Source	Background Strain	Other Information	Persistent ID / URL
**Parent – Male and Female**	Mus musculus	Alfred Nordheim	C57BL6\J	Srftm2.1Nor	MGI:2385465<br>Wiebel et al. Genesis 32, 124–126 (2002)
**Parent – Male and Female**	Mus musculus	Jacksons laboratory	C57BL6\J	Tg(Pdgfrb-cre/ERT2)6096Rha	RRID:IMSR_JAX:029684<br>https://www.jax.org/strain/029684
**Parent – Male and Female**	Mus musculus	Jacksons laboratory	C57BL6\J	Gt(ROSA)26Sor$^{tm4(ACTB-tdTomato,-EGFP)Luo}$	RRID:IMSR_JAX:007576<br>https://www.jax.org/strain/007576

Antibodies

Target antigen	Vendor or Source	Catalog #	Working concentration	Lot # (preferred but not required)	Persistent ID / URL
CD140a-FITC rat	Invitrogen	11-1401-82	1:100		RRID:AB_2572476
CD140b-APC rat	Invitrogen	17-1402-82	1:25		RRID:AB_1548743
CD144 rat	BD Biosciences	555289	1:100		RRID:AB_395707
CD31 goat	R&D Systems	AF3628	1:400		RRID:AB_2161028
CD31-PE rat	Biolegend	102408	1:50		RRID:AB_312903
CD34 rat	Abcam	ab8158	1:200		RRID:AB_306316
CD45-PB rat	Biolegend	103126	1:200		RRID:AB_493535
Cleaved-Caspase3 rabbit	Cell Signaling	9661	1:500		RRID:AB_2341188
ColIV rabbit	Bio-Rad	2150-1470	1:200		RRID:AB_2082660
Desmin rabbit	Abcam	ab15200	1:200		RRID:AB_301744
Endomucin rat	Abcam	ab106100	1:300		RRID:AB_10859306
ERG1 rabbit	Abcam	ab110639	1:100		RRID:AB_10864794
GFP goat	Abcam	ab6673	1:300		RRID:AB_305643
GFP-Alexa Fluor 488, rabbit	Invitrogen	A21311	1:200		RRID:AB_221477
Goat Alexa Fluor-488, donkey	Invitrogen	A32814	1:500		RRID:AB_2762838
Goat Alexa Fluor-647, donkey	Invitrogen	A21447	1:500		RRID:AB_2535864
ICAM-2, rat	BD Pharmigen	553326	1:200		RRID:AB_394784
NG2, rabbit	Millipore	AB5320	1:100		RRID:AB_91789
PDGFRβ, goat	R&D Systems	AF1042	1:100		RRID:AB_2162633
Rab Alexa Fluor-568, donkey	Invitrogen	A10042	1:500		RRID:AB_2534017
Rab Alexa Fluor-647, donkey	Invitrogen	A31573	1:500		RRID:AB_2536183

DOI [to be added]

Rabbit ab Alexa Fluor-488, donkey	Invitrogen	A21206	1:500		RRID:AB_2535792
Rab-HRP, goat	Cell Signaling	7074	1:10000		RRID:AB_2099233
Rat Alexa Fluor-488, donkey	Invitrogen	A21208	1:500		RRID:AB_141709
Rat Alexa Fluor-647, chicken	Invitrogen	A21472	1:500		RRID:AB_2535875
Sca1, rat	Abcam	ab51317	1:200		RRID:AB_1640946
Smooth muscle actin-Cy3, mouse	Sigma-Aldrich	C6198	1:300		RRID:AB_476856
Sox17, goat	R&D Systems	AF1924	1:100		RRID:AB_355060
SRF, rabbit	Cell Signaling	5147S	1:500		RRID:AB_10694554
Ter119, rat	BD Biosciences	553671	1:200		RRID:AB_394984
Ter119-PB, rat	Biolegend	116232	1:500		RRID:AB_2251160

DNA/cDNA Clones

Clone Name	Sequence	Source / Repository	Persistent ID / URL
MAL-GFP	N/A	Richard Treisman	Vartiainen and Guettler et al. *Science (80-)*. 2007;316:1749–1752

Cultured Cells

Name	Vendor or Source	Sex (F, M, or unknown)	Persistent ID / URL
Primary cultured pericytes	Primary isolates (mouse brain)	F,M	N/A
NIH/3T3	ATCC	unknown	CRL-1658
NIH/3T3-MAL-GFP	Richard Treisman	Unknown	Vartiainen et al. *Science (80-)*. 2007;316:1749–1752.

Data & Code Availability

Description	Source / Repository	Persistent ID / URL
Bulk-RNAseq dataset of sorted mural cells from *Srf*^{iMCKO} and control retinas	Mouse retina P12 / GEO	https://www.ncbi.nlm.nih.gov/geo/query/acc.cgi?acc=GSE205491

Other

Description	Source / Repository	Persistent ID / URL
0.05 % Trypsin-EDTA	Gibco	25300054
Albumin Fraction V (BSA)	Sigma-Aldrich	BSAV-RO
Cadaverine Alexa Fluor-488	Invitrogen	A30676
CCG-203971	Sigma-Aldrich	PZ0162-5MG
Click-iT EdU Cell Proliferation Kit Alexa Fluor-647	Thermofisher	C10340
Collagen Type 1	Corning	354249
Color Prestained Protein Standard (Broad Range)	New England Biolabs	P7719S
Donkey Serum	Sigma-Aldrich	S30-100ML
Dual-Luciferase Reporter Assay	Promega	E1910
Dulbecco's Modified Eagle Medium (DMEM)	Gibco	41965039
Dulbecco's Phosphate Buffered Saline	Sigma-Aldrich	D8537
EGM-2 Bulletkit	Lonza	CC-4176

DOI [to be added]

Endothelial Cell Basal Medium-2 (EBM-2)	Lonza	CC-3156
FastStart Universal SYBR Green Master (ROX) (2x conc.)	Roche/Sigma-Aldrich	4913914001
Fetal Calf/Bovine Serum	Gibco	26140079
Fluoromount-G	SouthernBiotech/Thermofisher	00-4958-02
Gelatine	Sigma-Aldrich	G2500-100G
Glutaraldehyde (25%)	Sigma-Aldrich	G5882-10X1ML
Isolectin B4, biotin conjugate	Sigma-Aldrich	L2140-1MG
Latex beads 2µm, carboxylatemodified polystyrene, fluorescent yellow-green	Sigma-Aldrich	L4530-1ML
Opti-MEM™ I Reduced Serum Media	Gibco	31985062
Papain Dissociation System	Worthington Biochemical Corporation	NC9067191
Paraformaldehyde (PFA)	Sigma-Aldrich	158127
Peanut oil	Sigma-Aldrich	P2144
Penicillin Streptomycin	Gibco	15140122
Pericyte Medium	Science Cell	1201
Platelet-Derived Growth Factor-BB human	Sigma-Aldrich	P3201-10UG
RNeasy Micro Kit	QIAGEN	74034
SiR-actin	Spirochrome	SC001
Streptavidin Alexa Fluor-405	Invitrogen	S32351
Streptavidin Alexa Fluor-488	Invitrogen	S11223
Tamoxifen	Sigma-Aldrich	T5648
Technical Oxygen	Westfalen Gas	/
TransIT-LT1	Mirus Bio	MIR2304

DOI [to be added]
