# Supplementary material for: Mural Cell SRF Controls Pericyte Migration, Vessel Patterning and Blood Flow
Source: Circ Res. 2022 Jul 14;131(4):308–27. doi: 10.1161/CIRCRESAHA.122.321109 (PMC9348820; doi:10.1161/CIRCRESAHA.122.321109)
Supplement: Supplementary file 10 [file res-131-308-s010.pdf]

SUPPLEMENTAL MATERIAL

Mural cell SRF controls pericyte migration, vessel patterning and blood flow

Michael M. Orlich[1,2,5], Dr. rer. nat.; Rodrigo Diéguez-Hurtado[6,7], PhD; Regine Muehlfriedel[3], Dr. rer. nat.; Vithiyanjali Sothilingam[3], Dr. rer nat.; Hartwig Wolburg[4], Dr. rer. nat.; Cansu Ebru Oender[1], M. Sc., Pascal Woelffing[1]. B. Sc.; Christer Betsholtz[5], PhD; Konstantin Gaengel[5], Dr. rer. nat.; Mathias Seeliger[3], Dr. med. Dipl.-Ing., Ralf H. Adams[6,7], Dr. phil. nat.; Alfred Nordheim[1,2], Dr. rer. nat.

Affiliations:

(1) Department of Molecular Biology, Interfaculty Institute for Cell Biology, University of Tuebingen, Tuebingen, Germany
(2) International Max Planck Research School (IMPRS) "From Molecules to Organisms", Tuebingen, Germany
(3) Institute for Ophthalmic Research, Centre for Ophthalmology, University Clinic Tuebingen (UKT), Tuebingen, Germany
(4) Department of General Pathology and Pathological Anatomy, Institute of Pathology and Neuropathology, University Clinic Tuebingen (UKT), Tuebingen, Germany
(5) Department of Immunology, Genetics and Pathology, Rudbeck Laboratory, Uppsala University, Uppsala, Sweden
(6) Department of Tissue Morphogenesis, Max Planck Institute for Molecular Biomedicine, Muenster, Germany
(7) Faculty of Medicine, University of Muenster, Muenster, Germany

Corresponding author:

Michael M. Orlich, michael.orlich@igp.uu.se, Rudbeck Laboratory C11, Dag Hammarskjölds Väg 20, 751 85 Uppsala, Sweden

Expanded Methods

Animal housing and ethical statement

Mice were kept in the animal facilities of the University of Tuebingen as well as the facilities of the University of Uppsala. Housing was performed under the national guidelines for research animal housing (GV-SOLAS, Germany) and in accordance with Swedish legislation and were approved by the local animal ethics committees prior to experimentation. Mice were held in type 2 long polycarbonate or in individually ventilated cages (IVCs, facilities Uppsala) in groups of 2-5 animals per cage. Conditions were a 12 h day/night cycle (6 a.m. to 6 p.m.), a relative humidity of 60 % and a temperature of 21 °C. Animals had unrestricted access to food (pellets, composition: crude protein 19.3 %, crude fat 3.3 %, crude fiber 4.4, crude ash 6.0, calcium 1.0, Phosphorus 0.7, low in phytoestrogens) and water ad libidum. New litters were weaned 3 weeks after birth and separated into new cages.

All animal experiments carried out in this work were performed in compliance with the relevant laws and guide lines of that apply to the University of Tübingen. Conducted animal experiments were approved by the local animal ethics committee and conducted with the permission by the regional authorities in Tübingen (Regierungspräsidium Tübingen), Germany (IM05/18G and IM02/19G) and in accordance with Swedish legislation, approved by the Uppsala Committee on the Ethics of Animal Experiments (C115/15 and C111515/16).

Mouse lines and inducible genetic experiments

Pdgfrb(BAC)-CreER^{T2} mice [21] were bred with *Srf-flex1* mice [20] carrying a floxed exon 1 (flex1) of the *Srf* gene to obtain *Pdgfrb-CreERT2::Srf-flex1* mice on a C57BL/6 genetic background. For recombination and high resolution microscopy analyses, the *Rosa^{mTmG}* reporter gene [23] was crossed into *Pdgfrb-CreERT2::Srf-flex1*. Postnatal gene deletion in newborn mice was induced via intragastric injection of 50 µg tamoxifen (Sigma, #T5648, dissolved in ethanol-peanut oil (#P2144)) on postnatal days (P) 1 to P3, respectively. In OIR experiments, animals were injected intraperitoneally (i.p.) with 200 µg of tamoxifen on 3 consecutive days between P12 and P14. To induce Cre-mediated recombination in adult animals (8 weeks old), 500 µg of tamoxifen was injected i.p. on 5 consecutive days. Both males and females were used for genetic experiments and are represented in an approximate 1:1 ratio.

Tissue preparation and Immunohistology

Retinas from postnatal mice were isolated for subsequent staining procedures. Whole eyes were fixed in 4 % paraformaldehyde (PFA, Sigma, #P6148) dissolved in phosphate buffered saline (PBS) for 2h either on ice (mild fixation for antibody (AB) staining) or at room temperature (RT; strong fixation for IB4 staining). Eye balls were washed in PBS followed by dissection of the retina. Afterwards, the retina was partially cut into four quadrants. Freshly prepared retinas were blocked and permeabilized in blocking solution containing PBS supplemented with 1 % BSA (Sigma, #A4378) and 0.5 % Triton X-100 (Sigma, #T8787) overnight at 4 °C. Primary AB (see **Table S1**) were dissolved in blocking buffer (supplemented with 2 % donkey serum (Sigma, #S30-M)) and incubated over night at 4 °C. The next day, the primary antibody solution was removed and retina samples were washed 5 times for at least 20 min with PBS. Next, samples were incubated with fluorescence-coupled secondary antibodies (see **Table S1**) for 2h at RT protected from light. After incubation, retinas were washed 5 times for at least 20 min with PBS. Finally, samples were mounted on a microscope slide using Fluoromount G (Southern Biotech, #0100-01) mounting medium.

When an isolectin-B4 (IB4; Sigma-Aldrich, #L2140) staining was performed, blocked Retina samples were washed thrice in Pblec buffer (1 µM $MgCl_2$, 1 µM $CaCl_2$, 0.1 µM $MnCl_2$ and 0.5 % TritonX-100 in PBS) and incubated with IB4 (see **Table S1**) overnight in Pblec at 4 °C. When a co-staining was necessary, primary antibodies were additionally added. The next day, IB4 stained tissues were washed 5 times for 20 min with PBS and subsequently incubated in fluorophore-coupled Streptavidin (see **Table S1**) together with additional secondary antibodies in blocking buffer for 2h at RT. Subsequent washing and mounting procedure were carried out as mentioned above. All mounted retina samples were stored protected from light at 4 °C.

In order to validate antibody specificity and distinguish genuine target staining from the background, primary and secondary antibodies were omitted in control experiments. All used secondary antibodies had been pre-absorbed by the manufacturer to minimize the cross-reactivity with non-specific targets.

Transmission electron microscopy

Eye balls were removed and retinas were dissected without any fixation. After dissection, the retinas were fixed in Karnowsky's fixative (2 % PFA, 2 % glutaraldehyde (Sigma, #G5882) solved in 0.1 M cacodylate buffer). Retinas were then washed in cacodylate buffer, post-fixed in 1 % osmium tetroxide (OsO4, Sigma, #201030) for 1h at RT and dehydrated in ascending series of ethanol (50, 70, 96, 100 %). In the 70 % step, ethanol was saturated with uranyl acetate (Honeywell Fluka, #73943) for contrast enhancement. Final dehydration was completed in propylene oxide. The specimens were embedded in Araldite adhesive (Serva). Semithin sections (1 µm) and ultrathin sections (50 nm) were generated on a FCR Reichert Ultracut ultramicrotome (Leica). Semithin sections were stained by using toluidine blue. Ultrathin sections were mounted on pioloform-coated copper grids, contrasted with lead citrate and analyzed and documented using an EM10A electron microscope (Carl Zeiss) running at 80 kV.

In vivo proliferation assay

For *in vivo* proliferation analysis of endothelial cells (ECs), thymidine analogue 5-Ethynyl-2´-deoxyuridine (EdU, Invitrogen, #10044) was prepared in DMSO-PBS (10:1, 2 mg/ml) and injected (15 µl/g of body weight) i.p. into P7 and P12 pups 3h (EC-proliferation) or 4 h (PC-proliferation) before sacrifice. Retinas were prepared and stained as described above. After secondary antibody staining, retinas were stained using the Click-it EdU Alexa Fluor-647 Imaging Kit (Invitrogen, #C10340) according to the manufacturer's instructions.

Oxygen induced Retinopathy (OIR)

The OIR disease model was performed as previously described [72], with slight modifications. In short, neonatal pups and their mothers were kept at normoxic conditions (room air) from birth to P7 to allow normal vascular development. For 5 days from P7 to P12, the mice were transferred to hyperoxic conditions of 75 % O_2. Finally, the animals were kept for another 5 days from P12 to P17 under normoxic conditions to induce the pathologic neovascular response. Hyperoxic conditions were realized by transferring the mice to a Biospherix A-Chamber (Biospherix) equipped with a ProOx 110 oxygen controller (Biospherix) connected to technical oxygen (99.99 % O_2). To reduce elevated CO_2 concentrations in the OIR chamber, 100 g of soda lime was kept at the bottom of the chamber as a quencher. Over the course of the experiment, oxygen concentration in the chamber was controlled

twice a day, while health status of the animals was controlled daily. To discharge the animals from the oxygen chamber, the oxygen concentration was decreased stepwise over a time course of 4h (1h of each 60 %, 50 %, 40 %, 30 % O_2). Tamoxifen injections to induce Cre-mediated recombination took place from P12 to P14 by 3 consecutive i.p. injections (see above). At P17, pups were sacrificed by exposure to CO_2 followed by cervical dislocation. Eye balls were removed and prepared as described above.

Fluorescence-activated cell sorting (FACS) of retinal pericytes (PCs)

FACs of retinal PCs was performed as previously described [7]. In short, eye balls from P12 pups were harvested and collected in ice cold MEM (Gibco, #31095-029) supplemented with 25 mM HEPES (Thermofisher, #15630049), penicillin/streptomycin (Gibco, #15140) and 10% fetal calf serum (FCS). Retinas were subsequently dissected and enzymatically digested using the Papain Dissociation Kit (Worthington Biochemical Corporation, #LK003150). After digestion, the single-cell suspension was filtered through a 40 µm cell strainer into a 2 ml reaction tube. Hereafter, the filtered cell suspension was centrifuged for 5 min at 300 x g at 4 °C and the supernatant was carefully removed. The remaining cell pellet was resuspended in 120 µl of FACS buffer (2% FCS and 2mM EDTA pH 8.0). 100 µl of the resuspended cells were mixed with 100 µl of FACS buffer containing fluorescently labeled ABs (Ter119-PB, CD45-PB, CD140a-FITC, CD140b-APC and CD31-PE, final dilutions see **Table S1**), followed by incubation for 30 min on ice. The remaining 20 µl of whole retina single-cell suspension was kept as an input control and lysed in 350 µl of RLT buffer plus (Quiagen) supplemented with β-mercaptoethanol (10 µl/ml). After AB incubation, 1 ml of FACS buffer was added to the cell suspension to wash out unbound ABs, followed by centrifugation for 5 min at 300 x g at 4 °C. Finally, the supernatant was carefully removed and the remaining cell pellet was resuspended in 500 µl FACS buffer containing 50 ng/ml DAPI (Sigma, #D9542) to label dead cells. Labeled single cell suspensions were sorted subsequently on a BD FACS Aria lllu sorter (BD Bioscience) using a 100 µm nozzle. Gating strategies for discrimination of cell populations were based on their immunolabelling defined according to reference experiments using single-stained samples and fluorescence minus one (FMO) controls.

Scanning-Laser Ophthalmoscopy (SLO) and optical coherence tomography (OCT)

The *in vivo* analysis of retinal layer structure was performed as published previously [73,74]. In short, mice were anesthetized with ketamine (66.7 mg/kg) and xylazine (11.7 mg/kg) and pupils dilated with tropicamide (Mydriaticum Stulln). OCT imaging was performed together with SLO on a commercially available system (Heidelberg Engineering Spectralis, Eye Explorer version 5.3.3.0). This device features a superluminescent diode at 870 nm as low coherence light source. OCT scans are acquired at a speed of 40.000 scans per second and each two-dimensional B-scan (set to 30° field of view) contains up to 1536 A-scans. We use black on white mode, i.e. high reflectivity is shown as dark and low reflectivity as white. For angiography, indocyanine green dye (ICG) was used as published [6,7] previously in combination with an infrared laser (795 nm; barrier filter 800 nm).

Intravital blood flow measurement

Intravital measurement of retinal blood flow was performed as previously described [48], with modifications. In short, mice were anesthetized with ketamine (66.7 mg/kg) and xylazine (11.7 mg/kg) and pupils dilated with 0.5% tropicamide (Alcon, Vnr#043182). Anesthetized animals were placed on

a heating pad and the eyes were moisturized with phosphate buffered saline (PBS). Afterwards, the tail vein was cannulated and microspheres were applied subsequently (see **Table S1**, 5 µl/g of body weight). The tail vein remained cannulated for the rest of the experiment. In order to adjust the refractive index of the mouse eye, a drop of 2.5% hypromellose (Sigma, #H3785) applied followed by a 5 mm glass cover slip. Next, the animal was placed under an upright Epi-fluorescence microscope (LEICA SP8 DIVE; Epi-fluorescence mode; Camera: CMOS Andor Zyla 4.2) and the head was positioned in order to enable a direct view to the retinal optic disk. Using a 10x objective (Nikon Ph1 DL, NA: 0.30, WD: 15.2 mm; objective adapter: Leica 505259) several series of time-lapse sequences were acquired using an exposure time of 8 ms. In order to visualize the entire vascular structure, a bolus of cadaverine (see **Table S1**, 5 µl/g of body weight) was infused via the tail vein while recording images. After imaging, the relevant tissues were taken and fixed as described above.

For the calculation of bead velocity, the streak length of 10 consecutive beads was measured in multiple veins, arteries and capillaries separately. Bead velocity was calculated by division of measured streak length, divided by the camera exposure time of 8 ms. In order to calculate approximate flow rates of arteries and veins, vessels were assumed to be cylindrical. For this, the diameter of cadaverine perfused arteries and veins was measured at three positions. Flow rates were calculated using following equation: $Flow\ [\frac{\mu m}{sec}] = \frac{v_{beads}\ [\frac{\mu m}{sec}] * \pi * d_{vessel}\ [\mu m]^2}{4}$.

Electroretinography (ERG)

ERGs were obtained as previously described [49]. Mice were dark-adapted overnight before the experiments and their pupils were dilated. Anesthesia was induced by subcutaneous injection of ketamine (66.7 mg/kg), xylazine (11.7 mg/kg) and atropine (1 mg/kg). The Espion V6 equipment (Diagnosys LLC) was used for the ERG measurements. Single flash recordings were obtained both under scotopic (dark-adapted) and photopic (light-adapted)) conditions. Light adaptation was accomplished with a background illumination of 30 cd/m^2 for 10 min, which stayed on during the course of the photopic recordings. Stimuli were presented with increasing intensities, reaching from 10–3 cd*s/m^2 to 30 cd*s/m^2, divided into 10 steps of 0.5 and 1 log cd*s/m^2. 10 responses were averaged with an inter-stimulus interval (ISI) of 5 s or 17 s (for 1, 3, 10 and 25 cd*s/m^2).

Western Blot

Western blot was performed to verify SRF ablation in cultured pBMCs at protein levels. pBMC cultures (control and *Srf-KO*) were grown to 70 % confluence on 6-well cell culture plates. Cells were trypsinized, pelleted and resuspended in ice cold RIPA buffer (50 mM Tris-HCl, 150 mM NaCl, 1 % TritonX-100, 0.5 % Sodium deoxycholate, 0.1 % SDS, 1 mM EDTA, pH 8.0), followed by agitation for 30 min at 4 °C. Subsequent to agitation, resuspended cells were centrifuged for 30 min at 10,000 x g at 4 °C. The supernatant containing proteins was then transferred into a fresh tube and the protein concentration was determined by a Bradford assay (Biorad, #5000201). Protein lysates were stored at -20 °C until used. 20 mg of protein were separated by SDS-PAGE and subsequently transferred onto a 0.22 µm polyvinylidene difluoride transfer membrane using a Bio-Rad transfer system for 50 min (2 mA /cm^2 of membrane). Following the transfer, membranes were washed in TBST buffer (138 mM NaCl, 20 mM Tris, 0.1% Tween-20 in H_2O, pH 7.4) and subsequently blocked in blocking buffer containing 5% nonfat died milk in TBST for 1h at RT. After blocking, membranes were incubated with the primary AB (see **Table S1**) diluted in blocking buffer overnight at 4°C. The next day, the membranes were washed 3 times with TBST and subsequently incubated with corresponding horse radish peroxidase (HRP)

coupled secondary AB (see **Table S1**) diluted in blocking buffer for 1h at RT. Finally, membranes were washed three times with TBST followed by a washing step with TBS (138 mM NaCl, 20 mM Tris in H_2O, pH 7.4) to remove detergents from the membrane. Imaging of membranes was performed by addition of chemiluminescent HRP substrate (Biorad, #1705061). Documentation was performed on a Fusion SL documentation system (Vilber, version V.070).

Luciferase assay

NIH/3T3 cells (ATCC CRL-1658) were transfected with SRF-reporter plasmids $(TSM)_2$ and $(TMM)_2$ [28] using the TransIT-LT1 (Mirus Bio LLC, #MIR 2300) transfection reagent. Afterwards, the cells were kept in starvation media (DMEM+0.3 % FCS), or in starvation media containing MRTF inhibitor (CCG-203971, 30 µM; Sigma, #SML1422). The following day, cells were stimulated as indicated by addition of Platelet-Derived Growth Factor-BB (PDGFBB; 25 ng/µl; Sigma, #SRP3138) for 7h. Afterwards, media was removed and lysates for luciferase assay were prepared according to the manufacturer's instructions (Promega, #E1910). Luciferase activity was measured using an OPTIMA FluoroSTAR (BMG LABTECH) or a Biotek Synergy HT microplate reader.

Isolation and culture of primary brain pericytes (pBPCs)

pBPCs were isolated from 6 to 8 weeks old C57BL/6 mice which harbor the homozygous *Srf-flex1* allele and a heterozygous *Rosa26*^{mTmG} allele. The isolation procedure was performed as previously described [75] with minor changes. In short, brains of 3 mice were dissected and placed in ice cold MEM media containing 1% pen/strep. Using a razor blade, the olfactory bulb, cerebellum and medulla were removed. The remaining cortical tissues were pooled and rigorously minced using a razor blade. Minced brains were washed using MEM and centrifuged for 5 min at 300 x g at RT. The tissue pellet was enzymatically digested using the Papain Dissociation Kit (Worthington Biochemical Corporation, #LK003150) according to the manufactures protocol at 37°C for 70 min. After incubation, the digested tissue was homogenized by slow trituration, passing it 10 times through a 18G needle, followed by passing it 5 times through a 21G needle. The homogenate was then resuspended in 5 ml of 22 % BSA/PBS solution and centrifuged at 1,300 x g for 10 min to separate the cell content in the pellet from the myelin in the upper phase. The supernatant was removed completely by aspiration and the cell pellet was then washed in EBM-2 media (Lonza, #CC-3162) followed by centrifugation at 300 x g for 5 min. After aspiration of the supernatant, the pellet containing single cells and vascular micro vessels was resuspended in 6 ml EBM2-medium and seeded equally in 3 wells of a 6- well plate coated with 0,2 % collagen type I (CollI; Corning, #CB354249). Plated cells were incubated at 37 °C and 5 % CO_2 for 20h in a humidified incubator to ensure attachment of viable cells to the plate. After incubation, the plates were carefully washed 3 times with 3 ml PBS. Finally, 3 ml of fresh EMB-2 media were added to each well and the cells were cultured until confluence. Cultures were then trypsinized (Gibco, #25200056), pooled and further cultured in a T25 cell culture flask. The established cell culture contained endothelial cells (ECs), fibroblasts and PCs, was cultured in EBM-2 medium until the second passage. pBPC culture, was established by culture the cells in PC medium (Sciencell, #1201) from the third passage onwards, which especially promotes mural cell growth. From passage 5 onwards, pBPC cultures were judged to have reached high purity [11]. Expression of mural cell markers was determined by qPCR. Cell cultures were also tested for mycoplasma contamination.

Tat-Cre transduction of pBPCs

For Tat-Cre transduction, 5 x 10^4 cells/6-well or 2 x 10^4 cells/24-well of pBPCs were seeded on 0.2% Coll coated plates and cultured overnight. The next day, cells were washed with PBS followed by addition of OptiMEM (Thermofisher, # 31985062) reduced serum medium. Then, Tat-Cre (Merck, #SCR508) was added to the wells (final concentration 4 µM) and incubated for 20h. The next day, the cells were carefully washed with PBS and recovered in PC-medium for 2 days. After recovery time, cells were directly used for experiments. The recombination efficiency was estimated by counting of eGFP positive cells compared to total cell count. Furthermore, *Srf* gene expression was determined by qPCR and western blot analysis.

Wound closure migration assay

Srf-KO and respective control pBPCs were trypsinized and counted as previously mentioned. 10 x 10^4 cells were seeded to 4-well plates of a 0.2 % Coll-coated 35 mm µ-Dish (Ibidi, #80466). Cells were cultured overnight in PC-medium and the next day, the culture insert was removed to generate wound-like gaps. The dish was washed with PBS and PC-medium containing 20 ng/ml PDGFBB (Sigma, #SRP3138), 750 nM SiR-Actin (Spirochrome, #SC001) as well as 1 µM verapamil was added. The stained cells were imaged for 20h at 37°C using a LSM800 (Zeiss) microscope at 10x magnification (PApo 10x/0.45) with 1 image cycle/10 min.

Trans-well migration assay

Srf-KO and respective control pBPCs were trypsinized and counted. Afterwards, the cells were resuspended in starvation media (basal PC-medium supplemented with 0.3% FCS) and 4 x 10^4 cells were seeded into a Boyden chamber insert with 8 µm pore size placed in a 24-well cell culture plate. Cells were incubated for 2h at 37°C and to let cells adhere. Then, the inserts were transferred to a new well containing 600 µl starvation medium supplemented with 20 ng/ml PDGFBB (Sigma, #SRP3138). Plates were incubated for 6h at 37°C to allow cells to migrate towards the gradient. Migration was stopped by replacement of the medium and subsequent fixation of the membranes using 4% of PFA for 10 min at RT. After washing of the membrane with PBS, cells were incubated in DAPI (3 µg/ml) to stain the nucleus of migrated cells. Membranes were subsequently washed 3 times with PBS. To remove not migrated cells, the inner part of the membrane was cleaned by a cotton swab. Finally, the membrane was cut out of the chamber and mounted on a microscope slide. Samples were stored at 4°C until imaged by fluorescence microscopy.

RNA-Seq analysis

RNA of FAC-sorted cells was isolated using the RNeasy micro plus kit (Quiagen, #74034) according to the manufacturer's instructions and assessed on a 2100 BioAnalyzer (Agilent technologies). RNA-Seq libraries were generated with the Single Cell/Low Input RNA Library Prep Kit (New England biolabs, #E6420L) and sequenced on an Illumina device. The raw sequencing files were obtained in FASTQ format and quality controls were assessed by using FASTQC (https://www.bioinformatics.babraham.ac.uk/projects/fastqc/). Obtained reads were aligned to the mouse genome assembly (GRCm38.p6 Ensembl release 92) using TopHat2 (version 2.1.1), following by generation of gene expression counts by using HTSeq-count (version 0.6.1) with the option -m intersection-

nonempty. Final gene expression analysis across the measured samples was performed by using DESeq2 package on protein-coding genes. A principal component analysis (PCA) was used to assess the overall similarity between the samples and performed based on transformed read counts. Differentially expressed genes (DEGs) were selected by using a false discovery rate p-value cut-off 0.5. Gene enrichment analysis (GSEA) of DEGs was performed using the GSEA-tool (version 4.0.3) and a cut-off p-value of <0.05. The dataset is available under the GEO repository (GSE205491; https://www.ncbi.nlm.nih.gov/geo/query/acc.cgi?acc=GSE205491)

Imaging and Morphometric analysis of retinal vasculature

Imaging of stained tissues was performed either on an Olympus FluoView 1200 confocal system (Objectives: UPlanSApo 10x/ 0.4; UPlanSApo 20x/ 0.75; : UPlanSApo 40x/ 0.95; UPlanSApo 60x/ 1.2 water Light-source (laser)/detector: DPSS (405 nm), Argon (488 nm/515 nm), DPSS (635 nm), GaAsp-PMT detectors) or a Zeiss LSM 800 confocal system (objectives: PApo 10x/0.45; Plan-Neofluar 20x/0.5; C-Apochromat 40x/ 1.2 water FCS; Plan-Apochromat 63x/1.4 oil; Light-source/detector: Lasermodule LM URGB, GaAsp-PMT detectors, Airyscan module for super resolution). Overview Images were taken on a Zeiss Axiozoom epifluorescence microscope system. For comparability of imaged tissues. Imaging of one experiment was performed exclusively on one of the above-mentioned systems. Laser intensity or exposure time was adjusted to control conditions and imaged using those parameters in one experiment.

Retinal vasculature microscopy images were analyzed using the ImageJ software tool Fiji (version 1.52p) [76]. For quantifications, the control group was set as reference and the mutant group was calculated accordingly. Images for quantifications were obtained in the most cases from two retinas out of one animal and were averaged and expressed as one value (n=1). In general, 8 (but at least 4) comparable fields of view were imaged per animal and averaged. Images for quantification were taken at high magnification (40x) and with a field of view covering 317 µm*317 µm. The number of utilized animals in each group is indicated in the respective figures. Vascularized area was measured as ICAM2$^+$, CD31$^+$ or IB4$^+$ area divided by total area. Radial outgrowth was measured as distance from the optic nerve to the periphery of the vascular front for each wedge of the retina. Endothelial filopodia at the angiogenic front were manually counted and normalized to a length of 1000 µm sprouting front. Branching points were manually counted and divided by the vascularized area. Mural cell coverage was determined by manually counting NG2$^+$ or PDGFRB$^+$ cells at the capillary plexus and the angiogenic front, normalized to the vascularized area. Mural cell coverage determined by DES staining was measured by DES$^+$ area divided by the total vascularized area. Empty sleeve area was measured as CollV$^+$ area subtracted by the IB4$^+$ vessel area, leaving only empty collagen sleeves. Empty collagen sleeve area was divided by IB4$^+$ vessel area. SMA signal intensity was measured by SMA intensity on arteries normalized to ICAM2 signal intensity. The background intensity of each assessed channel was subtracted from the respective channel intensity. SMA intensity was assessed by measurement of SMA signal intensity, subtracted by the background intensity. Extravasation of red blood cells was determined by subtraction of the vascularized area from the Ter119$^+$ erythrocyte area to detect extravasated red blood cells. Arteriovenous nicking (crossings of arterioles and venules) was counted manually on whole retina samples. EC density was determined by manually counting ERG$^+$ nuclei to specifically detect ECs and dividing through the ICAM2$^+$ vessel area. Levels of proliferating ECs were determined by counting of ERG$^+$/EdU$^+$ double-positive cells, normalized to ERG$^+$ cells. Arterial and venous diameter was measured on three independent positions within a distance of up to 1000 µm to the optic nerve and the individual diameter values were averaged. Vascular smooth muscle cell (vSMC) coverage was measured by manually counting eGFP$^+$ cells or measurement of NG2$^+$ or DES$^+$ area on

arteries and veins, normalized to the vascularized area. Avascular area of retinas in OIR experiments was measured by using the "polygon selection" tool and normalized to the vascularized area. NVT area was determined by manual selection of NVT areas using the affinity software (Serif; expressed in pixel values) normalized to the total area. PC and SMA coverage on NVTs was determined by measurement of the $NG2^+$ or DES^+ area normalized to the vascularized area.

Statistics

Statistical analyses were performed using the GraphPad Prism 9 software. All data if not otherwise stated are presented as mean ± standard deviation of the mean (s.d.). All experimental *in vivo* data are based on animal numbers (n). The used animal numbers (*in vivo*) or performed repetitions (*in vitro*) are annotated in the figures. When possible ($n \geq 6$), normal distribution of data was tested (if not otherwise stated) by using the Shapiro-Wilk test ($\alpha=0.05$). In case of normal distribution, the unpaired two-tailed Students t-test with Welch's correction was used ($\alpha=0.05$, confidence interval of 95%). For comparison of multiple groups (>2 groups), a one-way ANOVA followed by a Tukey's post hoc test was used. If data was uneven distributed or **n** was too small ($n<6$) the non-parametric Mann-Whitney test (two-tailed) was used to determine statistical significance. A *p-value*<0.05 was considered to be statistically significant. Blinding of samples was not performed and groups were randomly assigned. For determination of group sizes. No prior statistical group size determination was performed. Instead, group size was determined according to previous experience and reproducibility of the results across the independent experiments. No animals were excluded from the study. Representative images across the manuscript were chosen according to the most representative examples reflecting the typical phenotype.

Table S1. List of antibodies and staining reagents.

Antigen/staining reagent	Source, clone/number	Company	Dilution
NG2	Rabbit, AB5320	Millipore	1:100
Cadaverine Alexa Fluor-488	A30676	Invitrogen	NA
CD140a-FITC	Rat, APA5, 11-1401-82	Invitrogen	1:100
CD140b-APC	Rat, APB5, 17-1402-82	Invitrogen	1:25
CD144	Rat, 11D4.1 ,555289	BD Biosciences	1:100
CD31	Goat,AF3628	R&D Systems	1:400
CD31-PE	Rat, 390, 102408	Biolegend	1:50
CD45-PB	Rat, 30F-11, 103126	Biolegend	1:200
CollV	Rabbit, 2150-1470	Bio-Rad	1:200
Desmin	Rabbit, ab15200	Abcam	1:200
ERG1	Rabbit, ab110639	Abcam	1:100
Cleaved-Caspase3	Rabbit, #9661	Cell Signaling	1:500
Endomucin	Rat, ab106100	Abcam	1:300
Sox17	Goat, AF1924	R&D Systems	1:100
CD34	Rat, ab8158	Abcam	1:200
Sca1	Rat, ab51317	Abcam	1:200
GFP	Goat, ab6673	Abcam	1.300
GFP-Alexa Fluor 488	Rabbit, A21311	Invitrogen	1:200
ICAM-2	Rat,553326	BD Pharmigen	1:200
Isolectin B4	Bandeiraea simplicifolia	Sigma-Aldrich	1:25
Latex beads, carboxylate-modified polystyrene, fluorescent yellow-green	L4530-1ML	Sigma	NA
PDGFRβ	Goat, AF1042	R&D Systems	1:100
SiR-actin	SC001	Spirochrome	1:1000-1:3000
SMA-Cy3	Mouse,C6198, 1A4	Sigma-Aldrich	1:300
SRF	Rabbit, D71A9, 5147S	Cell Signaling Technology	1:500

Streptavidin Alexa Fluor-400	S32351	Invitrogen	1:100
Streptavidin Alexa Fluor-488	S11223	Invitrogen	1:100
Ter119	Rat,TER-199,553671	BD Biosciences	1:200
Ter119-PB	Rat, TER-119, 116232	Biolegend	1:200
α-rab HRP	Goat, 7074	Cell Signaling Technology	1:10000
α rat Alexa Fluor-488	Donkey, A21208	Invitrogen	1:500-1:1000
α-goat Alexa Fluor-488	Donkey, A32814	Invitrogen	1:500-1:1000
α-goat Alexa Fluor-647	Donkey, A21447	Invitrogen	1:500-1:1000
α-rab Alexa Fluor-488	Donkey, A21206	Invitrogen	1:500-1:1000
α-rab Alexa Fluor-568	Donkey, A10042	Invitrogen	1:500-1:1000
α-rab Alexa Fluor-647	Goat, A21244	Invitrogen	1:500-1:1000
α-rat Alexa Fluor-647	Chicken, A21472	Invitrogen	1:500-1:1000

Online Figures

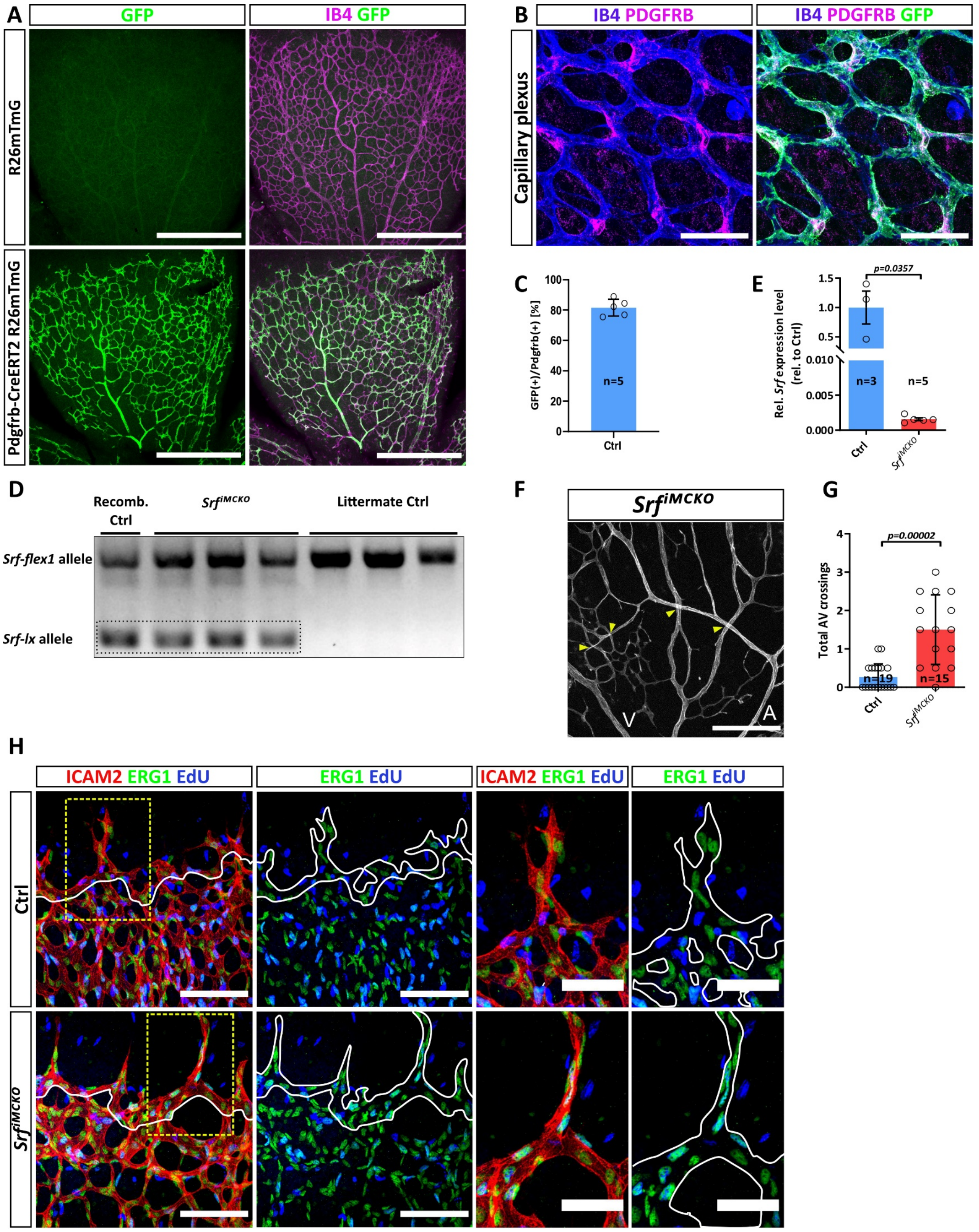

Figure S1| Verification of *Srf* deletion in retinal MCs and extended morphometric analyses. **(A)** Confocal images of tamoxifen induced *Pdgfrb-Cre*^{ERT2}::*Srf-flex1* heterozygous mice that carry the *Rosa26*^{mTmG} reporter . eGFP expression (green) in mural cells confirms the specificity of *Pdgfrb-Cre*^{ERT2}. Endothelial cells were co-stained with IB4. Scale bar, 500 µm. **(B)** High resolution confocal images of retinas stained for endothelial cells (IB4, blue), mural cells (PDGFRB, magenta) and recombined cells (eGFP, green), highlighting specific recombination in mural cells. Scale bar, 50 µm. **(C)** Quantification of the recombination rate of MCs. **(D)** Generic PCR of whole retina lysates from *Srf*^{iMCKO} mice showing the efficient recombination of the *flex1*-allele upon tamoxifen administration, resulting in a *Srf-lx* (exon 1 deletion) band. In littermate control mice, only the un-recombined *flex*1-allele becomes amplified **(E)** qPCR quantification of *Srf* expression levels of FACS sorted retinal MCs. (**F-G**) Confocal image and respective quantification of arteriovenous (AV)-nicking events (yellow arrowheads) in *Srf*^{iMCKO} retinas ("A" indicates artery and "V" vein). Scale bar, 200 µm. **(H)** Extended figure of figure 1(J) showing confocal images of proliferating cells (EdU, blue), endothelial cell apical surface (ICAM2, red) and endothelial nuclei (ERG1, green). White lines indicate the border of the leading front (first column) or the vessel shape (second and forth column). Images on the right panels show magnification of the yellow dashed squares shown on the left panel. Scale bar, 100 µm (left) and 50 µm (right). Error bars indicate s.d. of the mean. Statistical significance was determined using the unpaired Mann-Whitney test (two-tailed). Number of analyzed animals (n) is indicated.

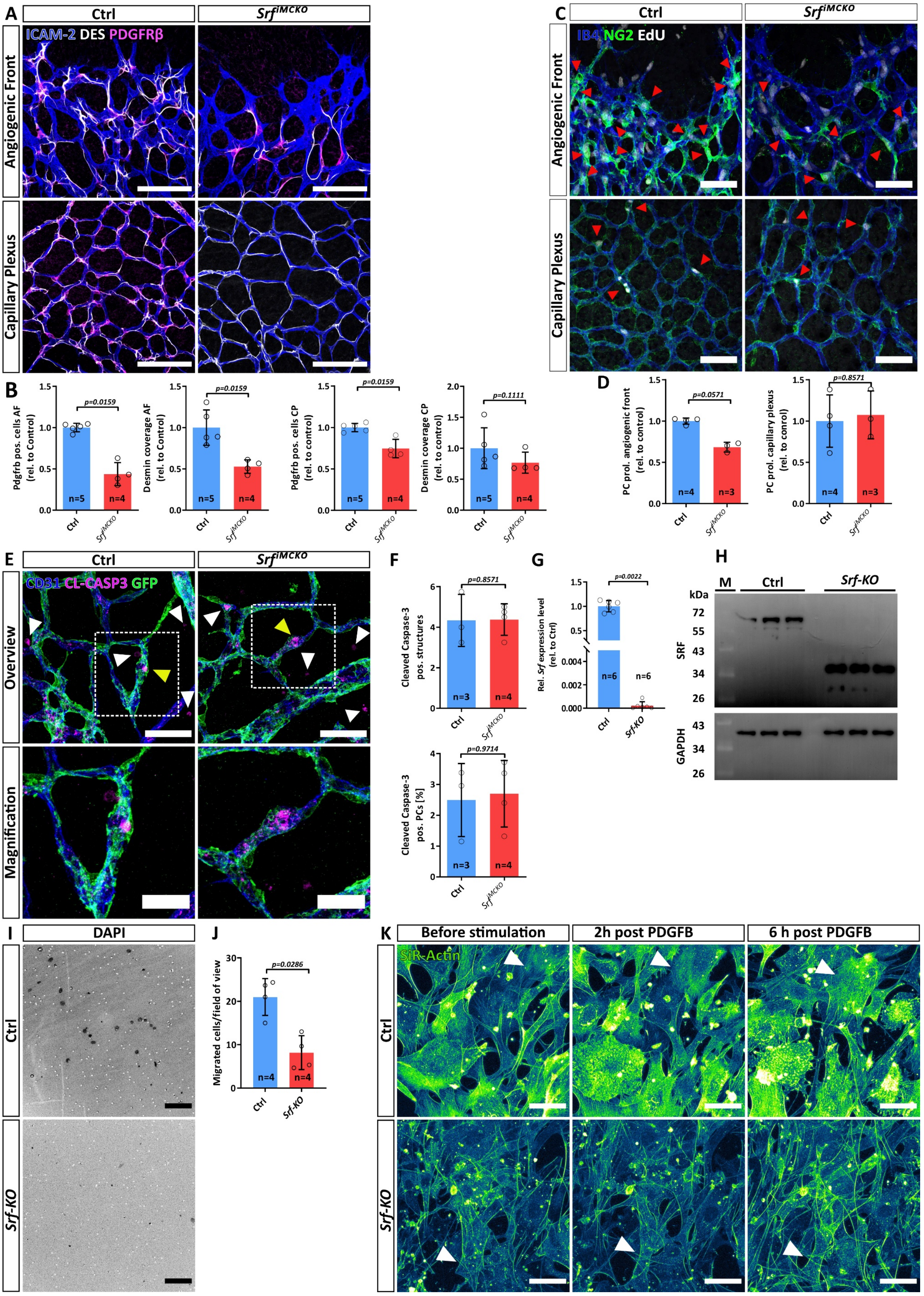

Figure S2| SRF-deficient pericytes (PCs) show migration defects. (A) Confocal images of PC coverage at the capillary plexus (CP) and the angiogenic front (AF). ECs were stained for ICAM2 (blue) and PCs for desmin (white) and PDGFRB (magenta). Scale bar, 100 µm. **(B)** Respective quantifications of the PC coverage. **(C)** Confocal images of proliferation events by EdU staining. Staining of the vasculature by IB4 and PCs by NG2. Red arrowheads point on proliferating PCs. Scale bar, 50 µm. **(D)** Respective quantification of PC proliferation. **(E)** Confocal images of apoptosis events visualized by staining of cleaved

Caspase-3 (Cl.-Casp3). Co-stained for CD31 (ECs) and GFP (*Rosa*^{mTmG} reporter). Arrowheads point on apoptotic cells (white) or apoptotic PCs (yellow). Scale bar, 50 µm (overview) and 25 µm (magnification). **(F)** Respective quantification of PC apoptosis events and total amount of apoptotic cells. **(G, H)** Verification of genetic *Srf* deletion in Tat-Cre treated *Srf-flex1* primary brain pericytes (pBPCs) via qPCR and western blot analysis, respectively. **(I)** Confocal images of a trans-well assay to determine polarized migration of pBPCs towards a PDGFB gradient trough a membrane with defined pore size. DAPI-positive dots (black) indicate migrated cells, which passed the membrane. Scale bar, 100 µm. **(J)** Respective quantification of the average of migrated cells per field of view. **(K)** Confocal images of a time-lapse experiment of stimulation of control and *Srf-KO* pBPCs with PDGFB. Note the accumulation of F-actin in control cells (white arrowheads, control panels), whereas the *Srf-KO* cells show no detectable accumulation of F-actin (white arrowheads, *Srf-KO* row). Scale bar, 50 µm. Error bars show s.d. of the mean. Statistical comparison by unpaired Mann-Whitney test (two-tailed). Number of analyzed animals/repetitions (n) is indicated.

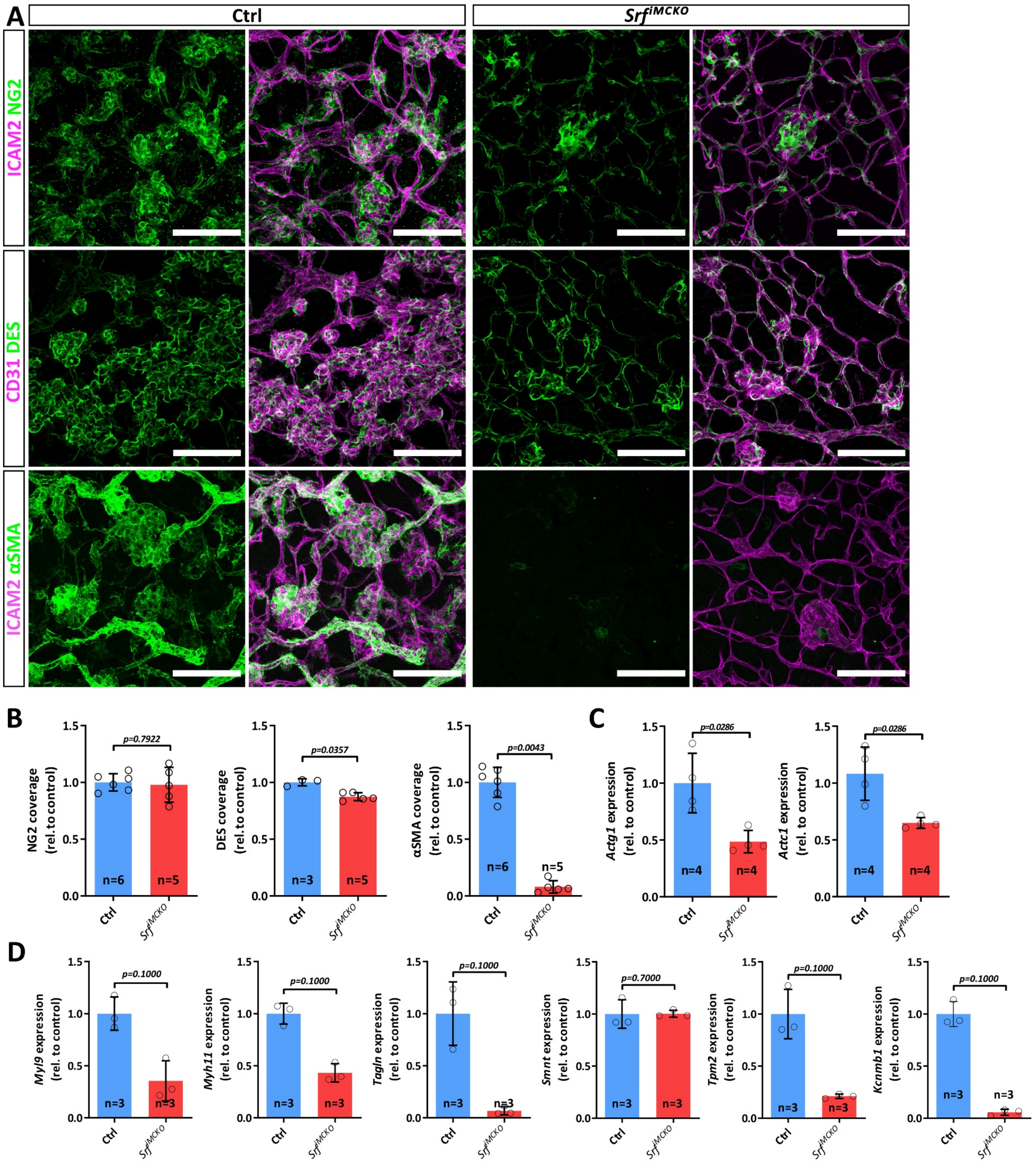

Figure S3| SRF is a driving force of pathologic pericyte (PC) activation. (A) Confocal images of control and *Srf*^{iMCKO} OIR retinas showing neovascular tufts (NVTs). Staining of NG2 (green, first row) or DES (green, second row) indicate PCs covering ICAM2 (magenta, first row) or CD31 (magenta, second row) stained vessels. Note the loss of αSMA signal (green, third row) as well as the reduction of amount and size of neovascular tufts (NVTs) in *Srf*^{iMCKO} OIR retinas. Scale bar, 100 µm. **(B)** Quantification of PC coverage of NVTs, using NG2 and DES as markers and of pathologically PC activation determined by αSMA coverage. **(C)** Quantification of relative transcript levels of muscle genes *Actc1* and *Actg1* determined by qPCR from whole OIR retina lysates. **(D)** Quantification of relative transcript levels of smooth muscle genes (SMGs) *Myl9*, *Myh11*, *Tagln*, *Smnt*, *Tpm2* and *Kcnmb1* determined by qPCR from whole OIR retina lysates. Error bars indicate s.d. of the mean. Statistical comparison by unpaired Mann-Whitney test (two-tailed). Number of analyzed animals (n) is indicated.

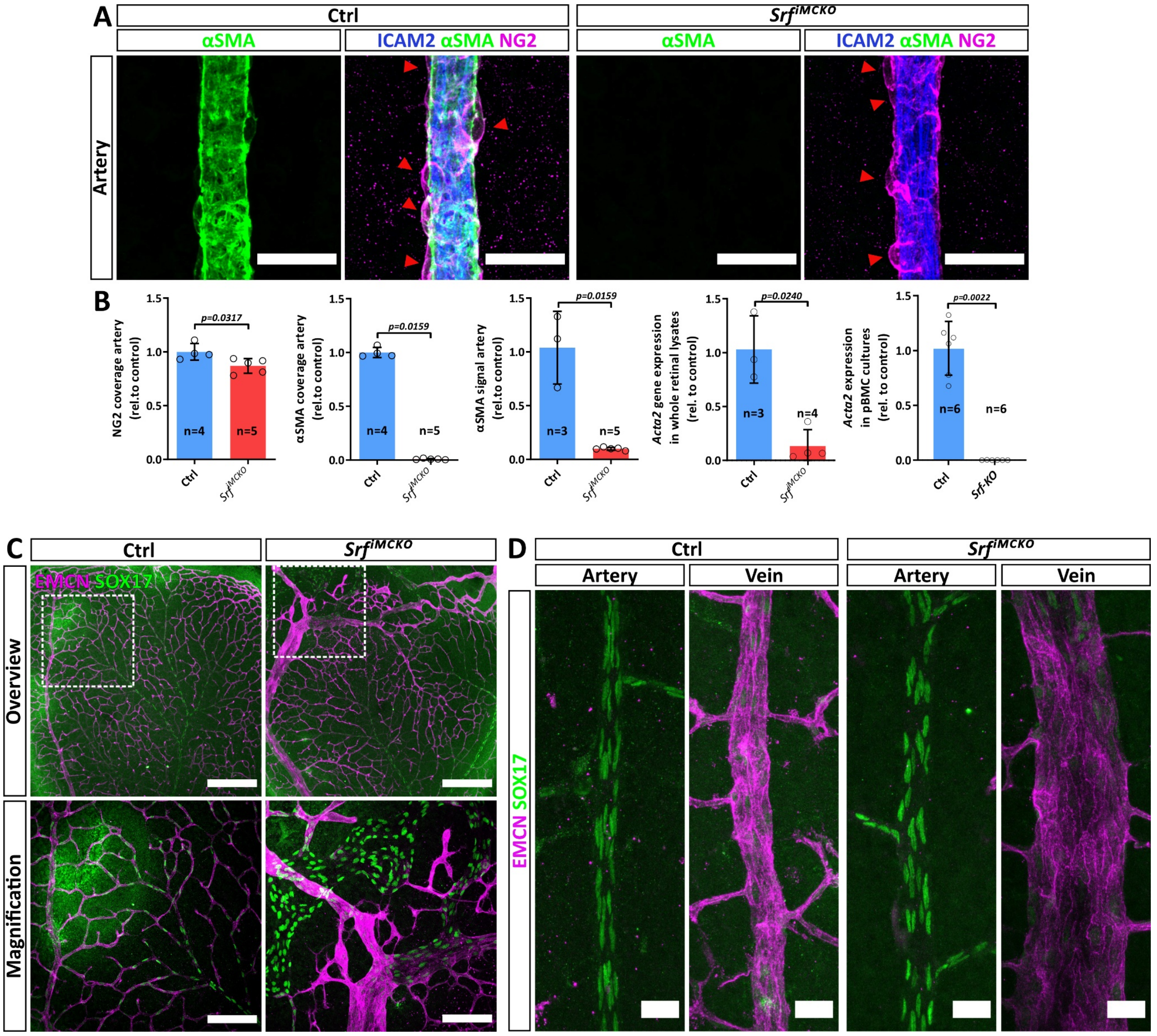

Figure S4| Expression of contractile markers and arteriovenous (AV) zonation in *Srf*^{iMCKO} retinas. (A) Confocal high-resolution images of P6 arteries, showing endothelial cells (ECs; ICAM2, blue), vSMC marker αSMA (green) and mural cell (MC) marker NG2 (magenta). Red arrowheads indicate colocalization of αSMA and NG2. Note the abolishment of αSMA in *Srf*^{iMCKO}. Scale bars, 25 µm. **(B)** Quantification of aSMC coverage determined by NG2 and αSMA coverage as well as by the measured intensity of αSMA staining. Relative transcript level of *Acta2* (encoding αSMA) determined by qPCR analysis of whole retina and pBPC culture lysates. **(C)** AV-zonation in P12 control and *Srf*^{iMCKO} retinas by staining of Sox17 (green, arterial EC identity) and Endomucin (EMCN, magenta, venous and capillary identity). Scale bar, 250 µm (overview) and 100 µm (magnification). **(D)** High resolution confocal image of AV-zonation of arteries and veins in control and *Srf*^{iMCKO} retinas. Scale, 25 µm. Error bars indicate s.d. of the mean. Statistical comparison by unpaired Mann-Whitney test (two-tailed). Number of analyzed animals/repetitions (n) is indicated.

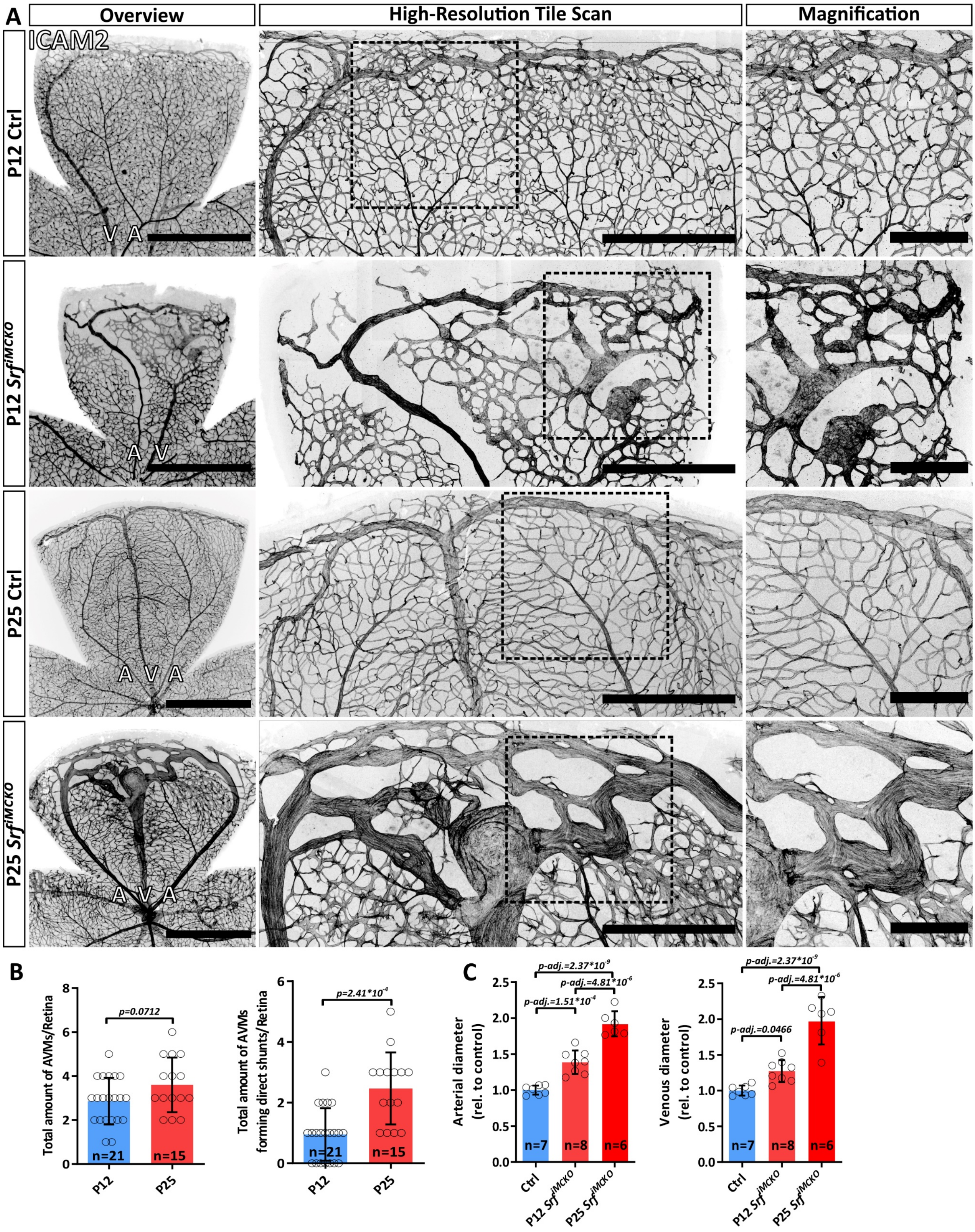

Figure S5| Arteriovenous malformations (AVMs) in *Srf*^{iMCKO} retinas worsen with increasing age. (A) Overview and high resolution confocal images of control and *Srf*^{iMCKO} retinas showing the retinal vasculature stained for the endothelial marker (ICAM2). Note the reduction of capillaries between the AV-shunts of P12 and P25 retinas. **(B)** Quantification of the total amount of AVMs per retina and total amount of AVMs forming a direct shunt without intercepting capillaries. **(C)** Quantification of arterial and venous diameter. Error bars indicate s.d. of the mean. Statistical significance in **B** (first graph) was determined using the Shapiro-Wilk test for normality followed by an unpaired t-test with Welch's correction (two-tailed). Data shown in **B** (second graph) were analyzed by the unpaired Mann-Whitney (two-tailed) test. For the data shown in **C**, comparison was performed using the Shapiro-Wilk test for normality followed by a one-way ANOVA multiple comparisons test with Tukey's-correction (3 comparisons). Number of analyzed animals (n) is indicated.

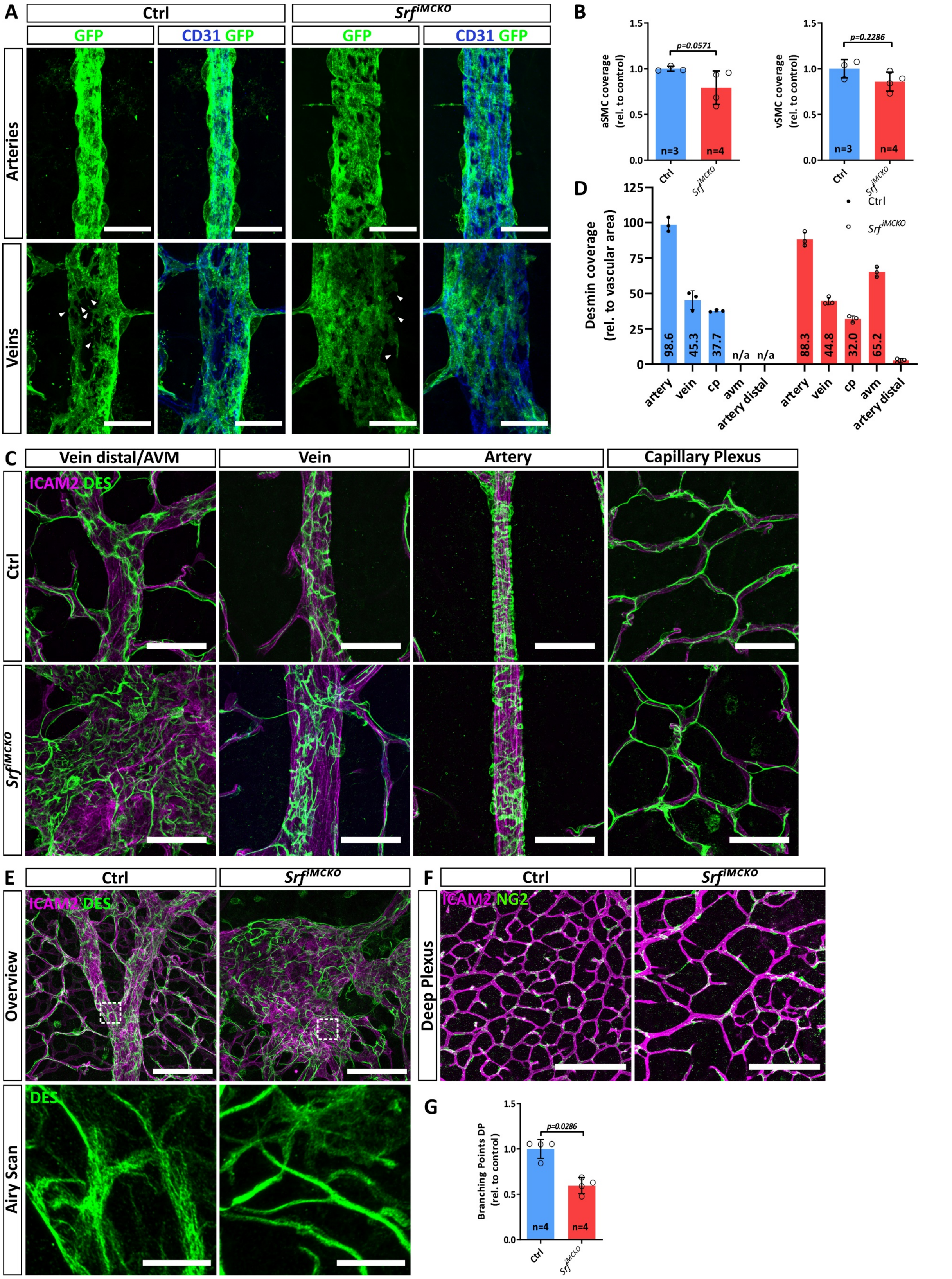

Figure S6| Altered morphology of vascular smooth muscle cells (vSMCs) on arteries and veins of *Srf*^{iMCKO} retinas at P12. (A) Confocal images of retinal arteries and veins from control and *Srf*^{iMCKO} mice bearing the *Rosa26*^{mTmG} Cre-reporter allowing GFP-tagging of mural cells (MCs; GFP, green) and co-stained for CD31 (endothelial cells, blue). Confocal high-resolution images of retinal arteries and veins. Scale bar, 25 µm **(B)** Quantification of arterial and venous SMC coverage, determined by cell counts per vascular area. **(C)** Confocal images showing representative MCs (desmin, green) coverage on vessels (ICAM2,

magenta). Scale bar, 50 µm. **(D)** Quantification of MC coverage (determined by desmin area/ICAM2 area). **(E)** Super resolution image (airy scan) of cytoskeletal desmin structure in control and *Srf*^{iMCKO} vSMCs. Scale bar, 100 µm (overview) and 10 µm (airy scan). **(F)** Vasculature of the deep plexus in P12 control and *Srf*^{iMCKO} retinas. Scale bar, 150 µm. **(G)** Quantification of branching points of the deep plexus. Error bars indicate s.d. of the mean. Statistical comparison by unpaired Mann-Whithey test (two-tailed). Number of analyzed animals (n) is indicated.

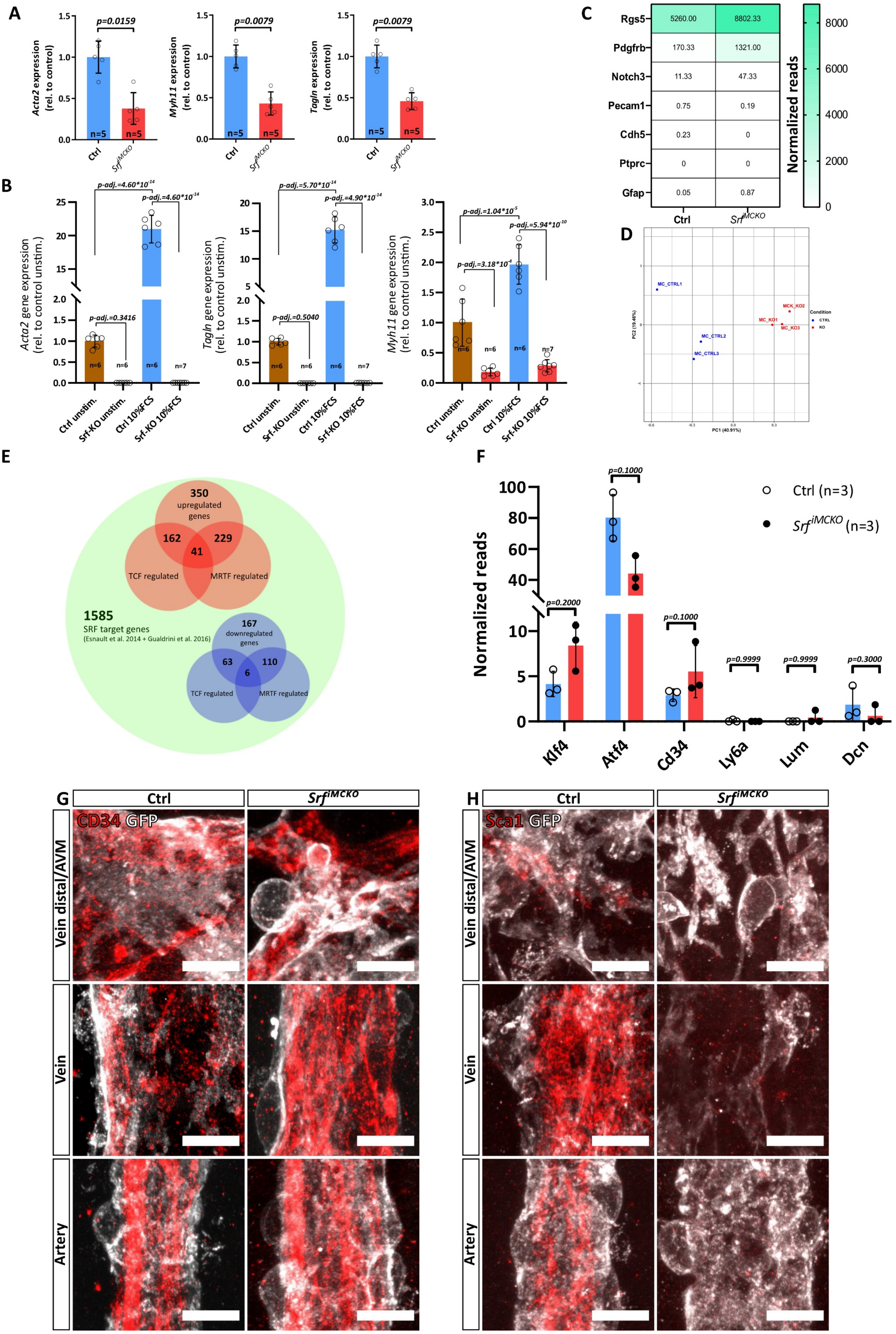

A
p=0.0159
Acta2 expression (rel. to control)
n=5
Ctrl
Srf iMCKO
p=0.0079
Myh11 expression (rel. to control)
p=0.0079
Tagln expression (rel. to control)
B
Acta2 gene expression (rel. to control unstim.)
p-adj.=4.60*10-14
p-adj.=0.3416
Ctrl unstim.
Srf-KO unstim.
Ctrl 10%FCS
Srf-KO 10%FCS
Tagln gene expression (rel. to control unstim.)
p-adj.=5.70*10-14
p-adj.=4.90*10-14
p-adj.=0.5040
Myh11 gene expression (rel. to control unstim.)
p-adj.=1.04*10-5
p-adj.=5.94*10-10
p-adj.=3.18*10-4
C
Rgs5 5260.00 8802.33
Pdgfrb 170.33 1321.00
Notch3 11.33 47.33
Pecam1 0.75 0.19
Cdh5 0.23 0
Ptprc 0 0
Gfap 0.05 0.87
Normalized reads
D
E
350 upregulated genes
162
229
41
TCF regulated
MRTF regulated
1585
SRF target genes
(Esnault et al. 2014 + Gualdrini et al. 2016)
167 downregulated genes
63
110
6
F
Normalized reads
Ctrl (n=3)
Srf iMCKO (n=3)
p=0.1000
p=0.2000
p=0.1000
p=0.9999
p=0.9999
p=0.3000
Klf4
Atf4
Cd34
Ly6a
Lum
Dcn
G
Ctrl
Srf iMCKO
CD34 GFP
Vein distal/AVM
Vein
Artery
H
Sca1 GFP

Figure S7| SRF deficient vascular smooth muscle cells (vSMCs) lose expression of contractile genes. (A) Gene expression of the vSMC markers *Acta2*, *Myh11* and *Tagln* in whole retina lysates. Error bars indicate s.d. of the mean. Statistical comparison by unpaired t-test with Welch's correction. Number of analyzed animals (n) is indicated. ns = not significant, $*p \leq 0.05$, $**p \leq 0.01$, $***p \leq 0.001$. **(B)** Transcription levels of vSMC genes *Acta2*, *Tagln* and *Myh11* measured by qPCR. Control and *Srf-KO* pBPCs were maintained in 2 % serum or stimulated with 10 % serum for 12h. **(C)** Heatmap of selected endothelial cell (EC), mural cell (MC), leucocyte and astrocyte marker expression across of isolated MCs measured by RNA-Seq. **(D)** Principal component analysis (PCA) plot of RNA-Seq data sets of control and Srf^{iMCKO} MCs. **(E)** Venn diagram summarizing upregulated and downregulated SRF target genes detected by RNA-Seq. The categorization of MRTF/TCF directed SRF targets are based on studies by Esnault et al. 2014 and Gualdrini et al. 2016. **(F)** Gene expression of markers, indicating differentiation of MCs towards a mesenchymal and fibroblast-like phenotype. **(G,H)** Confocal images of control and Srf^{iMCKO} retinal P12 arteries and veins labeled with the $Rosa26^{mTmG}$ reporter for MCs (GFP, white). Co-staining for differentiation marker SCA1 and CD34. Note that the signal is largely distributed in ECs. Scale bar, 10 μm. Statistical significance in **A** and **F** was determined by using the unpaired Mann-Whitney (two-tailed) test. Data shown in **B** were statistical compared using the Kolmogorov-Smirnov test for normality and a one-way ANOVA multiple comparisons test with Tukey's-correction (6 comparisons). Number of analyzed animals/repetitions (n) is indicated.

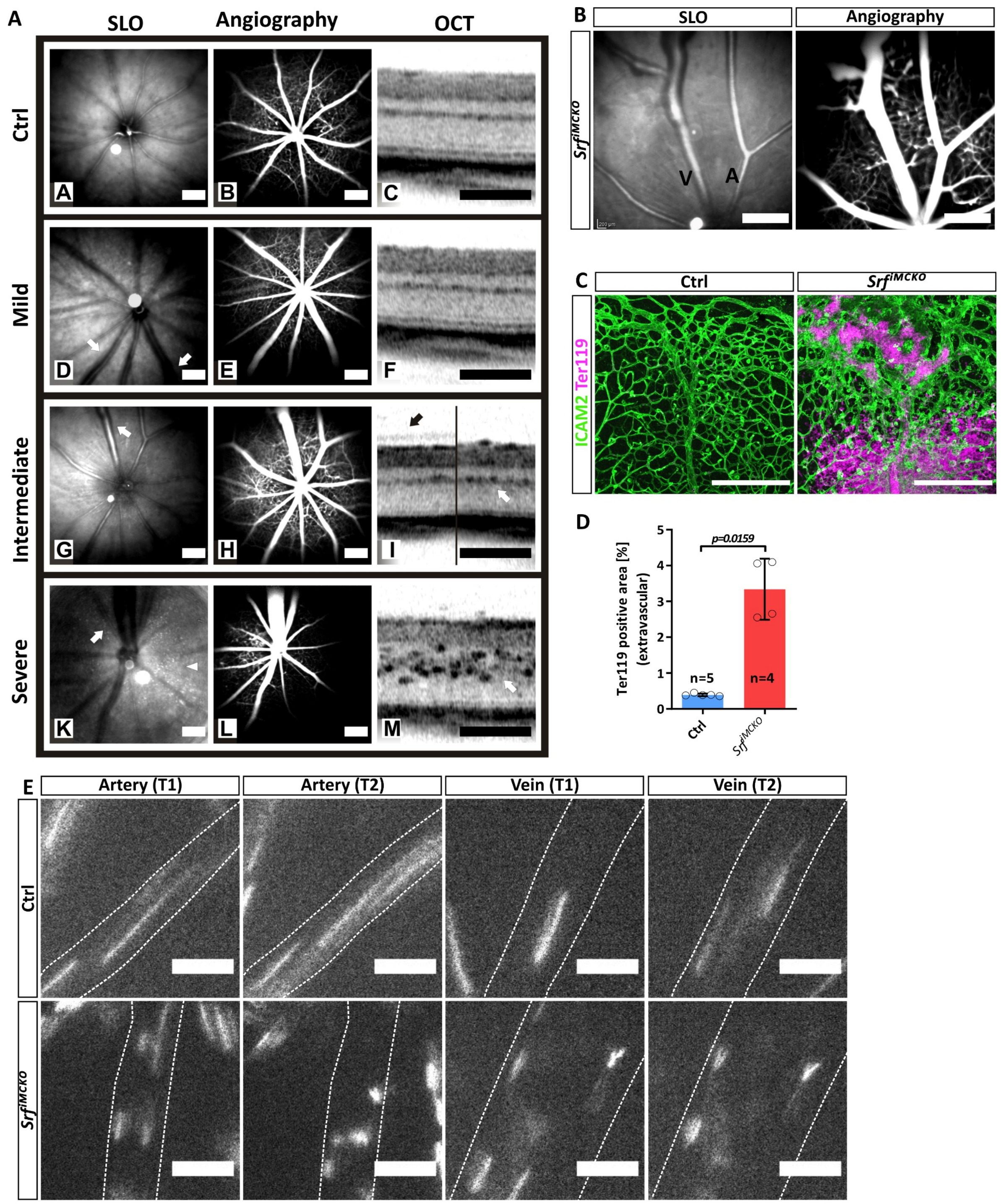

Figure S8| Intravital imaging of the retinal vasculature in control and *Srf*^{iMCKO} animals. (A) Extended version of Figure 7 (A) showing SLO, angiography and OCT images of 4 weeks old control and *Srf*^{iMCKO} live imaged retinas. Scale bar, 1 mm (SLO and Angiography panel) and 200 µm (OCT panel). **(B)** Higher magnification of *Srf*^{iMCKO} malformed vessels. Scale bar, 1 mm **(C)** Confocal images of P12 retinal vasculature (ICAM2, green) showing red blood cell (RBC) leakage. Scale bar, 250 µm. **(D)** Respective quantification of extravascular Ter119^{+} area as a measure for RBC leakage. **(E)** Intravital imaging of control and *Srf*^{iMCKO} retinal vasculature by imaging of fluorescent beads in the circulation. S Note the length of strokes indicates the bead velocity. Statistical comparison by unpaired Mann-Whitney test (two-tailed). Number of analyzed animals (n) is indicated.

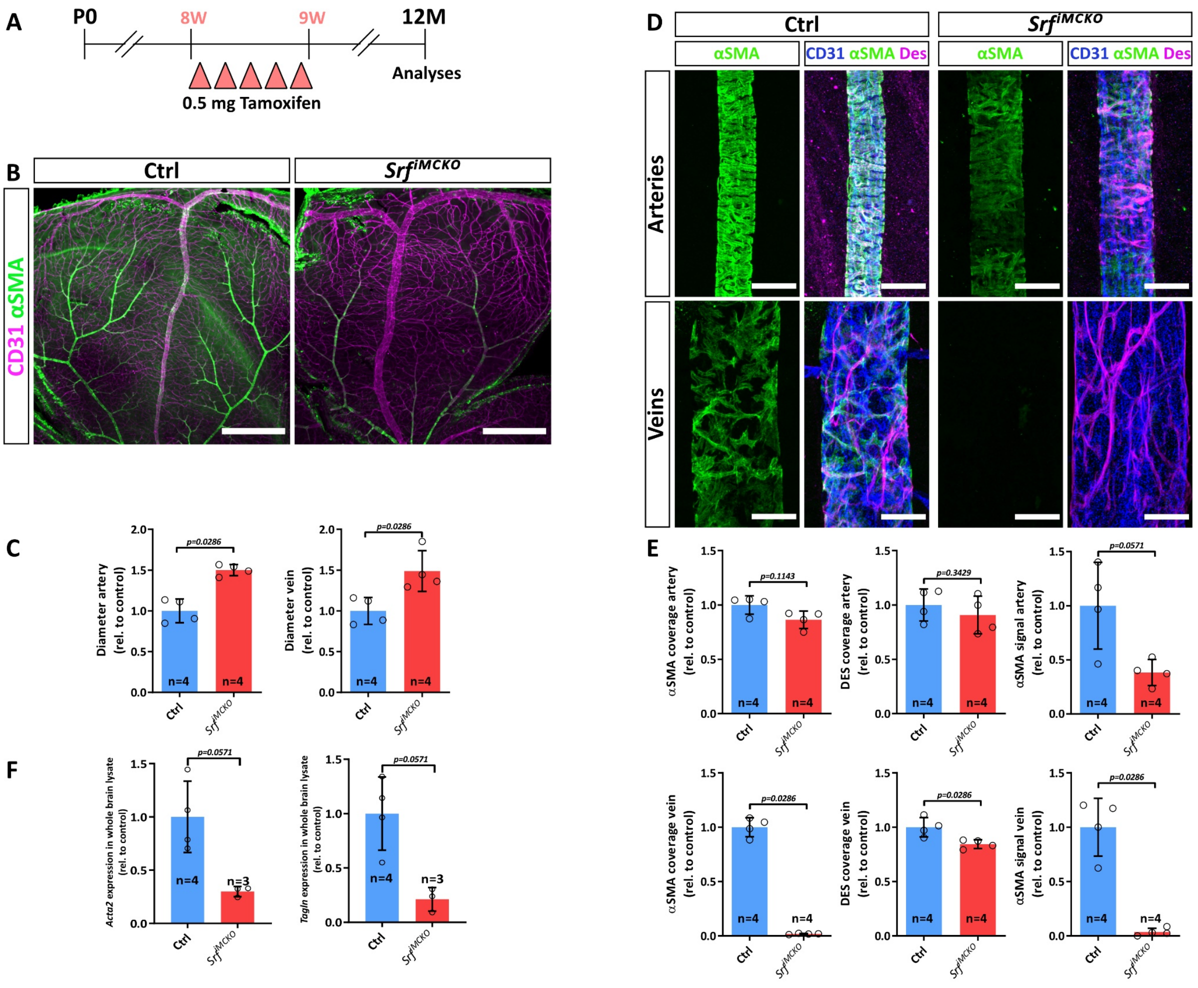

Figure S9| SRF is necessary for sustained contractile properties of vascular smooth muscle cells (vSMCs). (A) Illustration of the experimental approach to delete the *Srf* gene in mural cells of adult mice (8-weeks old) via tamoxifen administration at indicated timepoints. Mice were analyzed at the age of 12 months. **(B)** Overview images of the retinal vasculature of control and *Srf*^{iMCKO} retinas stained for CD31 and αSMA. Note the enlarged vessel diameter of arteries and veins as well as the reduced signal intensity of αSMA staining in *Srf*^{iMCKO} retinas. **(C)** Quantification of diameters of arteries and veins (upper graphs) as well as the quantification of the expression of vSMC marker genes *Acta2* and *Tagln* determined in whole brain lysates. **(D)** Confocal high-resolution images of arteries and veins stained for CD31, αSMA and DES. Note the strong reduction of αSMA signal in *Srf*^{iMCKO}. DES positive structures confirm the presence of vSMCs. **(E)** Quantification of αSMA and DES coverage as well as the αSMA signal intensity on arteries and veins. Error bars indicate s.d. of the mean. Statistical comparison by unpaired Mann-Whitney test (two-tailed). Number of analyzed animals (n) is indicated.

Online Videos

Video S1 |Representative migration assay of *Srf-KO* and control pBPC cultures stained by SiR-Actin. Cells were stimulated with PDGFB to stimulate migration. Note the difference in migration speed.

Video S2 |Representative PDGFB stimulation of *Srf-KO* and control pBPC cultures stained by SiR-Actin. Cells were stimulated with PDGFB to observe cytoskeletal modulation. Note the absence of reaction of *Srf-KO* pBPCs.

Video S3 |Representative PDGFB stimulation of 3T3 cells expressing MRTFA-GFP. 3T3 cells were stimulated with PDGFB to observe MRTFA-GFP nuclear translocation.

Video S4 |Representative SLO *in vivo* imaging of Srf^{iMCKO} and respective control retinas. Note the strong vessel motion of dilated vessels indicating a loss of vascular tone in Srf^{iMCKO} retinas.

Video S5 | Representative intravital microscopy of Srf^{iMCKO} and respective control retinas. Fluorescent beads (2 µm diameter) were injected to observe the blood flow in the retinal vasculature. Note the application a of a bead bolus starting in the middle of the video.

Video S6 | Representative intravital microscopy of Srf^{iMCKO} and respective control retinal capillaries. Fluorescent beads passing capillaries between arteries and veins. Note the reduction of bead crossings in Srf^{iMCKO} capillaries.
